# Design of peptide-containing *N*5-unmodified neutral flavins that catalyze aerobic oxygenations†
†Electronic supplementary information (ESI) available. See DOI: 10.1039/c7sc01933e


**DOI:** 10.1039/c7sc01933e

**Published:** 2017-05-30

**Authors:** Yukihiro Arakawa, Ken Yamanomoto, Hazuki Kita, Keiji Minagawa, Masami Tanaka, Naoki Haraguchi, Shinichi Itsuno, Yasushi Imada

**Affiliations:** a Department of Applied Chemistry , Tokushima University , Minamijosanjima , Tokushima 770-8506 , Japan . Email: imada@tokushima-u.ac.jp; b Institute of Liberal Arts and Sciences , Tokushima University , Minamijosanjima , Tokushima 770-8502 , Japan; c Faculty of Pharmaceutical Sciences , Tokushima Bunri University , Yamashiro , Tokushima 770-8514 , Japan; d Department of Environmental and Life Sciences , Toyohashi University of Technology , Toyohashi 441-8580 , Japan

## Abstract

Simulation of the monooxygenation function of flavoenzyme (**Fl-Enz**) has been long-studied with *N*5-modified cationic flavins (**FlEt^+^**), but never with *N*5-unmodified neutral flavins (**Fl**) despite the fact that **Fl** is genuinely equal to the active center of **Fl-Enz**. This is because of the greater lability of 4a-hydroperoxy adduct of **Fl**, **Fl_OOH_**, compared to those of **FlEt^+^**, **FlEt_OOH_**, and **Fl-Enz**, **Fl_OOH_-Enz**. In this study, **Fl** incorporated into a short peptide, flavopeptide (**Fl-Pep**), was designed by a rational top-down approach using a computational method, which could stabilize the corresponding 4a-hydroperoxy adduct (**Fl_OOH_-Pep**) through intramolecular hydrogen bonds. We report catalytic chemoselective sulfoxidation as well as Baeyer–Villiger oxidation by means of **Fl-Pep** under light-shielding and aerobic conditions, which are the first **Fl-Enz**-mimetic aerobic oxygenation reactions catalyzed by **Fl** under non-enzymatic conditions.

## Abstract 2

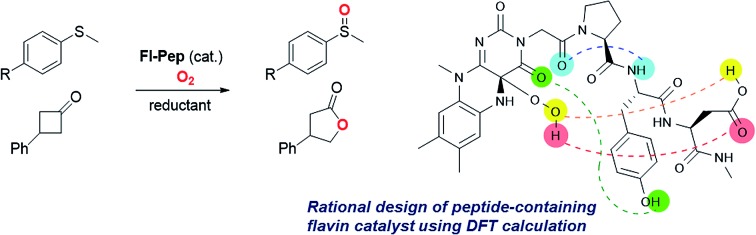
The first flavoenzyme-mimetic aerobic oxygenations catalyzed by *N*5-unmodified neutral flavin were realized with flavopeptides (**Fl-Pep**) rationally designed by computational calculations.

## Introduction

**Fig. 1:**
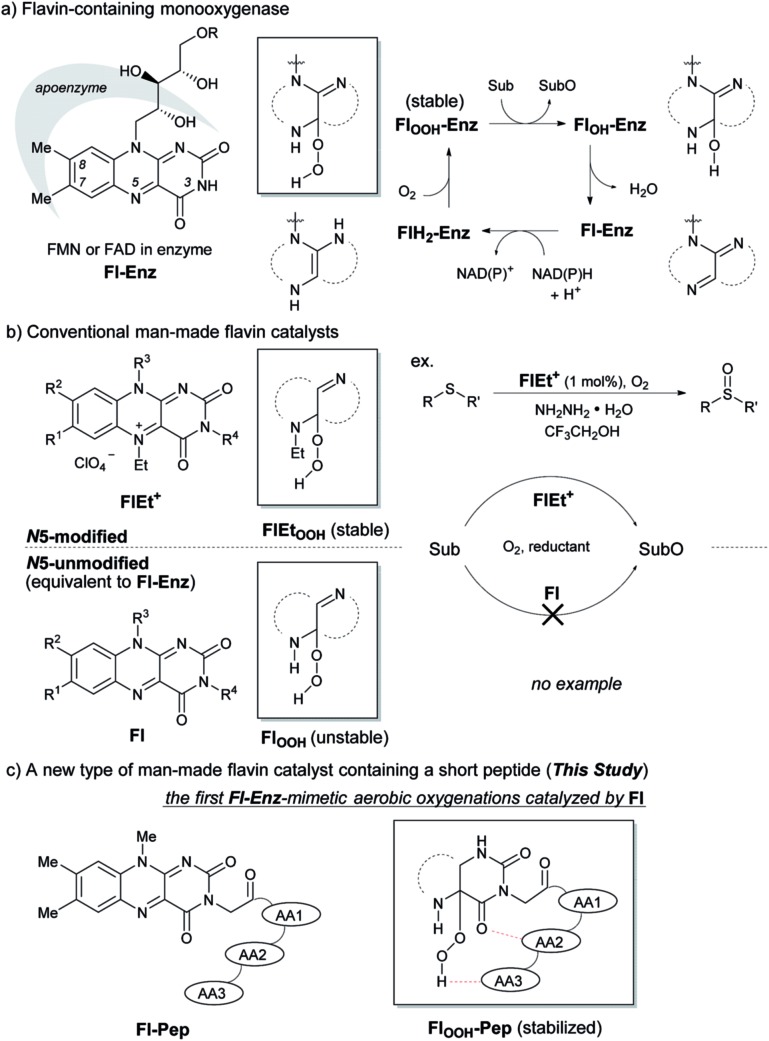
Flavin-catalyzed aerobic oxygenation reaction.

Isoalloxazines, such as riboflavin and its analogues (Fig. 1a), show flexible redox activities as well as visible-light emission properties due to the specific conjugated heterocyclic structure, which are responsible for the catalytic functions of a variety of flavoenzymes such as flavin-containing monooxygenase (**Fl-Enz**, Fig. 1a), oxidase, and photolyase.^1^ Whereas a number of flavin-inspired catalytic reactions for organic synthesis have been developed with artificial isoalloxazines, *N*5-modified cationic flavins (**FlEt^+^**, Fig. 1b, upper),^2^ there has been much less progress in developing those with genuine isoalloxazines, *N*5-unmodified neutral flavins (**Fl**, Fig. 1b, lower), under non-enzymatic conditions despite their availability and the fact that nature actually utilizes them as catalysts. Recently, **Fl** has received increasing attention because of its economical as well as environmental friendliness and appeared as thermal-redox,3a–c photoredox,3d–i and photosensitizing catalysts.3j–m However, the use of **Fl** as oxygenation catalysts simulating the function of **Fl-Enz** has remained unexplored.

The catalytic cycle of the oxygenation by **Fl-Enz** has been well understood (Fig. 1a) as a result of numerous early studies on flavin chemistry.^1^ A single oxygen atom is transferred from 4a-hydroperoxyflavin (**Fl_OOH_-Enz**), a key active species in **Fl-Enz** catalysis, to a substrate (Sub) to give an oxidized product (SubO) and 4a-hydroxyflavin (**Fl_OH_-Enz**), which eliminates H_2_O to form the oxidized flavin **Fl-Enz**. Then, **Fl-Enz** is reduced with NAD(P)H to afford the reduced flavin (**FlH_2_-Enz**), which finally reacts with molecular oxygen to regenerate **Fl_OOH_-Enz**. Previously, we have successfully simulated this catalytic cycle using **FlEt^+^** and hydrazine monohydrate instead of **Fl-Enz** and NAD(P)H, respectively (Fig. 1b, upper).^4^ For example, the aerobic oxygenation of sulfides was feasible in the presence of 1 mol% of 5-ethyl-3-methyllumiflavinium perchlorate (Fig. 1b, R^1^ = R^2^ = R^3^ = R^4^ = Me in **FlEt^+^**), 1 equivalent of hydrazine monohydrate, and 1 atm of O_2_ in 2,2,2-trifluoroethanol (TFE), in which TFE was crucial as a reaction solvent for predominant oxidation of sulfides in the coexistence of readily oxidizable hydrazine. By contrast, 3-methyllumiflavin (Fig. 1b, R^1^ = R^2^ = R^3^ = R^4^ = Me in **Fl**) was sluggish as a catalyst under the same reaction conditions, which was not surprising because of a kind of common knowledge that there is a huge difference in stability between the active species, 4a-hydroperoxyflavins **Fl_OOH_-Enz**, **FlEt_OOH_**, and **Fl_OOH_** (Fig. 1a and b). While **Fl_OOH_-Enz** can be properly stabilized by hydrogen bonds between its **Fl_OOH_** and peripheral proteins (**Enz**)^5^ and also **FlEt_OOH_** themselves are relatively stable,^6^ enzyme free **Fl_OOH_** are typically so labile and readily decomposed to H_2_O_2_ and **Fl**. In 1988, Tamao and co-workers introduced **Fl**-catalyzed aerobic Tamao–Fleming oxidation, in which the eliminated H_2_O_2_ from **Fl_OOH_** was utilized as an oxidant for the reaction.3*a* Very recently, König reported **Fl**-catalyzed oxidative chlorination of arenes under visible-light irradiation, in which the eliminated H_2_O_2_ from **Fl_OOH_** was utilized for converting acetic acid to peracetic acid that subsequently oxidizes Cl^–^ to OCl^–^, the active species for the chlorination.3*i* The only relevant work on **Fl_OOH_**-related oxygenation was reported by Yoneda and co-workers who showed that an artificial **Fl** bearing a carboxyl group at C6 position could promote the oxidation of thioanisole, although the oxidant was H_2_O_2_ and its actual active species was not identified.^7^ As a result, the development of **Fl-Enz** mimetic aerobic oxygenation catalyzed by **Fl** has never been realized.

Herein, we present the first **Fl**-catalyzed aerobic oxygenation reactions under non-enzymatic conditions. To break through this long-standing challenge, we envisioned **Fl** containing a short peptide such as di- or tripeptides, flavopeptide (**Fl-Pep**), which might be stabilized in its 4a-hydroperoxy adduct (**Fl_OOH_-Pep**) by intramolecular hydrogen bonds between **Fl_OOH_** and **Pep** (Fig. 1c). Though peptides as catalysts have recently become powerful tools for organic synthesis with the advancement of combinatorial “bottom-up” screening methods using peptide libraries, the rational “top-down” design of peptidic catalysts from a large degree of molecular diversity is still highly challenging.^8^ In this study, we successfully designed **Fl-Pep** as efficient catalysts for aerobic sulfoxidation as well as aerobic Baeyer–Villiger oxidation by a top-down approach that simply consists of computational estimation^9^ followed by experimental fine-tuning of suitable structures.

## Results and discussion

### Computational design and synthesis of **Fl-Pep**

**Fig. 2:**
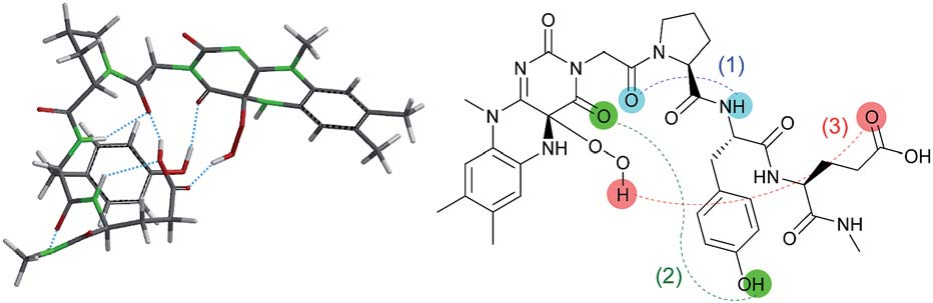
Lowest energy structure of 3-FlC2_4a(*R*)OOH_-Pro-Tyr-Glu-NHMe estimated by DFT calculation (left) and graphical representation of remarkable hydrogen bonds (right).

The design of **Fl-Pep** (Fig. 1c) was started by hypothesizing the following things: (i) **Pep** should be connected to the N3 position of **Fl** relatively close to the active site; (ii) readily available lumiflavin-3-acetic acid (3-FlC2)^10^ should be used as **Fl** and incorporated to the N terminus of **Pep**; (iii) a simple di- (AA1-AA2) or tripeptide (AA1-AA2-AA3) should be designed as **Pep** using inexpensive l-amino acids; (iv) l-proline residue should be placed at AA1 to induce constrained γ-turn structure and make the active site and AA2-AA3 spatially close; (v) AA2 and/or AA3 should be filled with acidic amino acid residues that can be expected to interact with the active site by intramolecular hydrogen bonds. In accordance with these design policies, we initially supposed 3-FlC2-Pro-AA2 and 3-FlC2-Pro-AA2-AA3 as the frameworks of **Fl-Pep**. To estimate appropriate structures for AA2/AA3 in **Fl-Pep**, lowest energy conformations of several **Fl_OOH_-Pep** bearing different amino acid residues in vacuum were explored by DFT calculation at B3LYP/6-31G* level. Stable conformations of dipeptidic **Fl_OOH_-Pep**, 3-FlC2_4a(*R*)OOH_-Pro-Glu-NHMe, 3-FlC2_4a(*R*)OOH_-Pro-Tyr-NHMe, and 3-FlC2_4a(*R*)OOH_-Pro-Gly-NHMe had no desirable intramolecular hydrogen bonds in calculation. On the other hand, tripeptidic 3-FlC2_4a(*R*)OOH_-Pro-Tyr-Glu-NHMe was suggested to be a promising sequence whose stable conformation includes ideal intramolecular hydrogen bonds between (1) CO neighboring to the nitrogen atom of Pro and NH of Tyr (γ-turn), (2) C(4)O of 3-FlC2 and OH in the side chain of Tyr, and (3) 4aOOH of 3-FlC2 and CO in the side chain of Glu (Fig. 2). Such a set of hydrogen bonds was not observed when Tyr-Glu in 3-FlC2_4a(*R*)OOH_-Pro-Tyr-Glu-NHMe was replaced with other residues, Phe-Glu, Asp-Glu, and Tyr-Ser. In addition, replacement of either Pro with β-Ala or 3-FlC2_4a(*R*)OOH_ with 3-FlC2_4a(*S*)OOH_ also led to loosing effective hydrogen bonds. These results obtained from just the above 9 calculation samples (for more details see ESI†) led us to synthesize **Fl-Pep** consisting of the sequence of 3-FlC2-Pro-Tyr-Glu.

The synthesis of **Fl-Pep** was accomplished by standard solid phase peptide synthesis following Fmoc/*t*Bu protocol using an amine-functionalized polystyrene resin (NH_2_-PS) (see ESI†).

### Aerobic sulfoxidation catalyzed by **Fl-Pep**

**Fig. 3:**
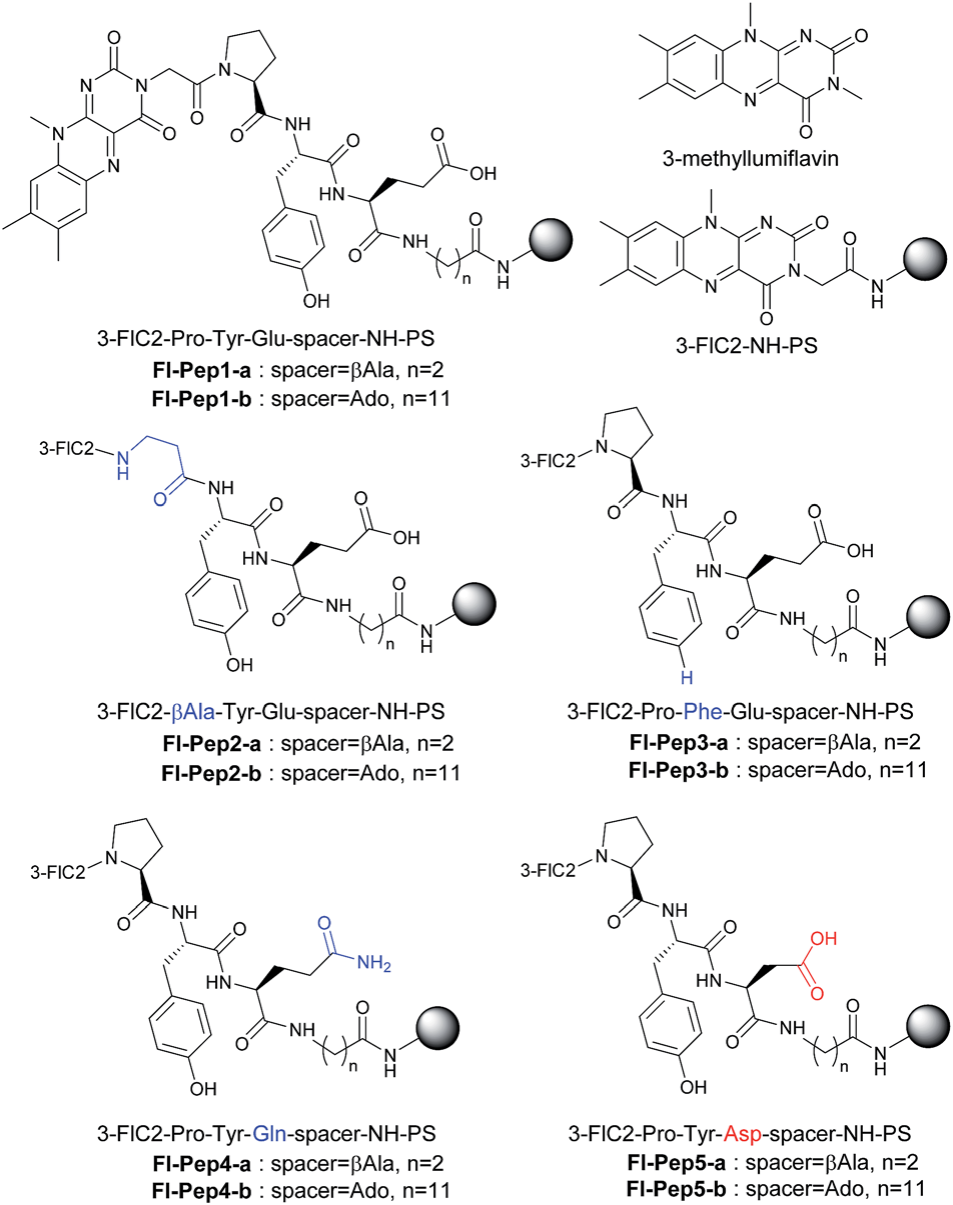
Structures of flavopeptides **Fl-Pep1–Fl-pep5**.

First of all, 3-FlC2-Pro-Tyr-Glu-βAla-NH-PS (**Fl-Pep1-a**, Fig. 3) bearing the peptide sequence designed by the above computational calculation was synthesized and tested as a polymer-supported peptide catalyst^11^ for aerobic oxidation of thioanisole under conditions that were previously developed by us for the reaction catalyzed by **FlEt^+^**.^4^ In the presence of 10 mol% of **Fl-Pep1-a** and 1 atm of O_2_ and 4 equivalents of hydrazine monohydrate in TFE, 9% of thioanisole was converted to methyl phenyl sulfoxide in 36 h (Table 1, entry 1), which was hopeful despite its low efficiency because the reaction did not proceed at all in the absence of the catalyst under otherwise identical conditions. As the efficiency of an insoluble polymer-supported catalyst can be strongly influenced by the nature of a reaction solvent,^12^ we subsequently used the mixed solvent of TFE and 1,2-dichloroethane (DCE) that can make polystyrene resin well swollen. As expected, the desired reaction was smoothly catalyzed by **Fl-Pep1-a** to give methyl phenyl sulfoxide in 60% yield in 36 h without any side reactions such as overoxidation to methyl phenyl sulfone (Table 1, entry 2). It should be noted that no reaction occurred in the absence of either TFE, O_2_, hydrazine (NH_2_NH_2_), or **Fl-Pep**, which indicates that all of them are essential. In addition, light was certainly shut out during the successful reaction, so that the involvement of singlet oxygen could be ruled out.3j,m Moreover, the excellent chemoselectivity, which is one of the feature of flavin catalyst,^1^,^2^ could leave the participation of peracid out and suggest **Fl_OOH_-Pep** as a major oxidant. Furthermore, 3-methyllumiflavin as well as 3-FlC2-NH-PS was ineffective as a catalyst under the same reaction conditions (Table 1, entries 3 and 4), showing the Pro-Tyr-Glu sequence in **Fl-Pep1-a** is responsible for its catalytic activity.

**Table 1:** Flavopeptide-catalyzed aerobic oxidation of thioanisole^*a*^

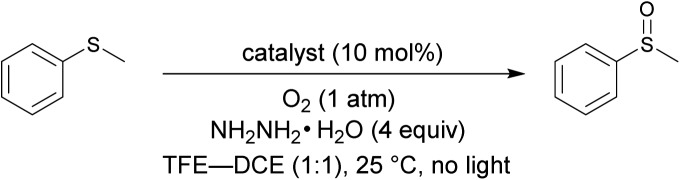			
Entry	Catalyst	Time (h)	Yield^*b*^ (%)
1^*c*^	**Fl-Pep1-a**	36	9
2	**Fl-Pep1-a**	24	36(60)^*d*^
3	3-Methyllumiflavin	36	2
4	3-FlC2-NH-PS	36	1
5	**Fl-Pep2-a**	36	10
6	**Fl-Pep3-a**	36	25
7	**Fl-Pep3-a** + 10 mol% phenol	36	18
8	**Fl-Pep4-a**	36	15
9	**Fl-Pep4-a** + 10 mol% AcOH	36	16
10	**Fl-Pep5-a**	24	52(78)^*d*^
11	**Fl-Pep1-b**	24	44
12	**Fl-Pep2-b**	24	18
13	**Fl-Pep3-b**	24	18
14	**Fl-Pep3-b** + 10 mol% phenol	24	16
15	**Fl-Pep4-b**	24	12
16	**Fl-Pep4-b** + 10 mol% AcOH	24	10
17	**Fl-Pep5-b**	24	62(99)^*d*^

^*a*^Reactions were performed using 0.1 mmol of thioanisole, 0.4 mmol of hydrazine monohydrate in 0.5 ml of a mixture of TFE and DCE (1 : 1) in the presence of 10 mol% of the catalyst under 1 atm of O_2_ at 25 °C.

^*b*^Determined by GC analysis.

^*c*^In TFE.

^*d*^Value after 36 h.

To explore structural and functional requirements for the catalytic activity of **Fl-Pep1**, we synthesized some analogues **Fl-Pep2–Fl-Pep5** (Fig. 3) and compared their catalytic activity with **Fl-Pep1** in the aerobic oxidation of thioanisole (Table 1). When Pro was replaced with βAla (3-FlC2-βAla-Tyr-Glu-βAla-NH-PS, **Fl-Pep2-a**), the catalytic activity dropped considerably (entry 5). Likewise, the replacement of Tyr with Phe (3-FlC2-Pro-Phe-Glu-βAla-NH-PS, **Fl-Pep3-a**), and that of Glu with Gln (3-FlC2-Pro-Tyr-Gln-βAla-NH-PS, **Fl-Pep4-a**) led to large decreases in reaction efficiency, respectively (entries 6 and 8), which were not improved even if a catalytic amount of phenol (entry 7) or acetic acid (entry 9) was used as an external additive. Interestingly, by contrast, enhancement of activity was observed (entry 10) when Glu was replaced with Asp (3-FlC2-Pro-Tyr-Asp-βAla-NH-PS, **Fl-Pep5-a**). These results indicate that the structure and functionality of all amino acid residues initially designed by the computational method was crucial for the efficient catalysis and, in particular, the carboxylic acid functionality of AA3 could play a significant role for fine-tuning of the activity. The same tendency on catalytic activities of **Fl-Pep1–Fl-Pep5** was observed by using those immobilized on polystyrene resin having a longer alkyl spacer (**Fl-Pep1-b–Fl-Pep5-b**, Fig. 3) with rather better performance (entries 11–17), probably because both conformational flexibility of the immobilized **Fl-Pep** and its accessibility to the substrate are enhanced. The best efficiency was achieved with **Fl-Pep5-b** for the present reaction, which provided methyl phenyl sulfoxide in 99% yield in 36 h (entry 17).^13^ It should be noted that all reaction yields in Table 1 were determined by GC analysis without product isolation to precisely evaluate the catalytic activity of each **Fl-Pep**.^14^ In addition, no methyl phenyl sulfone was observed in any cases.

**Fig. 4:**
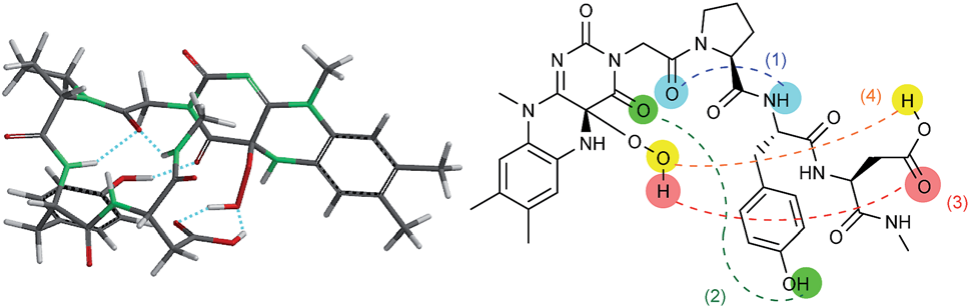
Lowest energy structure of 3-FlC2_4a(*R*)OOH_-Pro-Tyr-Asp-NHMe estimated by DFT calculation (left) and graphical representation of remarkable hydrogen bonds (right).

With these results in hand, we revisited the computational prediction of **Fl-Pep** to ensure its validity. We calculated 3-FlC2_4a(*R*)OOH_-Pro-Tyr-Gln-NHMe, which was proven to be an ineffective sequence (Table 1, entries 8 and 15), and FlC2_4a(*R*)OOH_-Pro-Tyr-Asp-NHMe, which was found to be the most effective sequence (Table 1, entries 10 and 17). In accordance with the experimental results, an effective set of hydrogen bonds (1), (2), and (3), similar to that highlighted in Fig. 2, were observed only in the lowest energy structure of FlC2_4a(*R*)OOH_-Pro-Tyr-Asp-NHMe (Fig. 4, for others see ESI†). It seems obvious that the Asp-derivative (Fig. 4) has an even better coordination than the Glu-derivative (Fig. 2) between the carboxylic acid and the hydroperoxy moiety with an additional interaction (4).

**Fig. 5:**
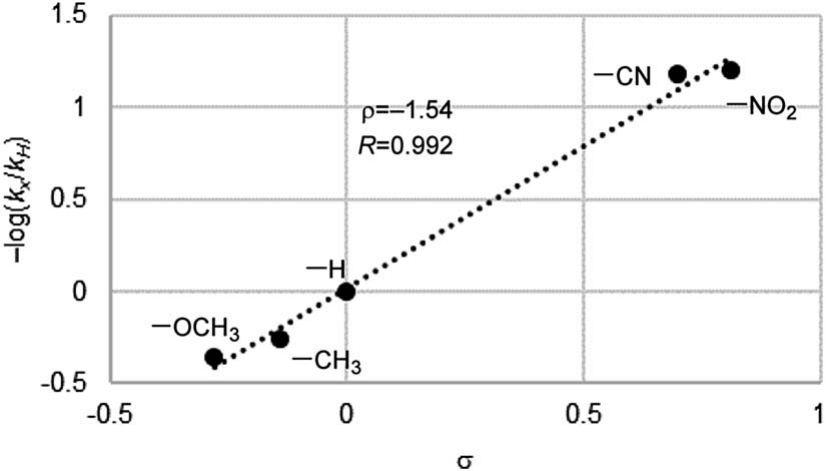
Hammett plot for aerobic oxidation of *p*-substituted methyl phenyl sulfides catalyzed by **Fl-Pep1-a**.

To gain an insight into active species for the oxygen transfer, we performed a Hammett study for the present aerobic sulfoxidation using **Fl-Pep1-a**. The relative reactivity values for *p*-substituted thioanisoles with respect to X = H (*k*_X_/*k*_H_) were determined, and the corresponding –log(*k*_X_/*k*_H_) *versus* the Hammett *σ* values were plotted to give *ρ* value of –1.54 (Fig. 5). The *ρ* value is similar to that of the stoichiometric oxidation of sulfides with **FlEt_OOH_** (*ρ* = –1.47)^15^ and also those of the aerobic (*ρ* = –1.60)4*b* as well as H_2_O_2_ sulfoxidation (*ρ* = –1.90)4*b* catalyzed by **FlEt^+^**. This result suggests that the present oxidation of sulfides takes place electrophilically with **Fl_OOH_-Pep** as the active species.

### Aerobic Baeyer–Villiger oxidation catalyzed by **Fl-Pep**

Encouraged by the above results we turned our attention to the Baeyer–Villiger oxidation for expanding the utility of **Fl-Pep** catalyst. Previously we developed this type of reaction catalyzed by **FlEt^+^**, which has so far been the sole example of organocatalytic Baeyer–Villiger oxidation using O_2_ as a terminal oxidant.^16^ The active species of nucleophilic **FlEt_OOH_** generated *in situ* allowed for selective Baeyer–Villiger oxidation of cyclobutanones into the corresponding γ-butyrolactones in the presence of alkene or sulfide functionality that could be readily oxidized with mCPBA, a typical oxidant for Baeyer–Villiger oxidation. Thus, **Fl-Pep** has also a great potential in the development of aerobic Baeyer–Villiger oxidation, and such chemoselectivity will be a strong evidence for the involvement of **Fl_OOH_-Pep** as an active species.

The Baeyer–Villiger oxidation of 3-phenylcyclobutanone into β-phenyl-γ-butyrolactone was used as a test reaction under conditions that were previously developed by us for the reaction catalyzed by **FlEt^+^**.^16^ In the presence of 5 mol% of **Fl-Pep5-b**, 1 atm of O_2_, 20 equivalents of H_2_O, and 3.5 equivalents of zinc dust in a mixed solvent of acetonitrile, toluene, and ethyl acetate (8 : 4 : 1), the desired oxidation proceeded smoothly to afford the target product in 72% yield in 7 h (Table 2, entry 1).^17^ Ethanol can be used instead of both CH_3_CN as a hydrophilic co-solvent and water as an essential proton source,^16^ however, toluene was crucial as a hydrophobic co-solvent that could render polystyrene resin properly swollen (see ESI†). As expected, 3-methyllumiflavin (entry 2) as well as 3-FlC2-NH-PS (entry 3) was totally inactive under the same reaction conditions. These results convinced us that, as in the case of the above sulfoxidation, an appropriate peptide sequence in **Fl-Pep** is essential for the catalysis involving the key stabilization of **Fl_OOH_-Pep** as illustrated in Fig. 4.

**Table 2:** Flavopeptide-catalyzed aerobic Baeyer–Villiger oxidation^*a*^

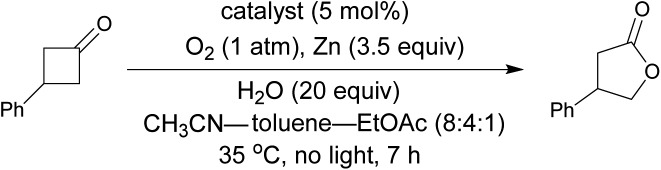		
Entry	Catalyst	Yield^*b*^ (%)
1	**Fl-Pep5-b**	72
2	3-Methyllumiflavin	<1
3	3-FlC2-NH-PS	2

^*a*^Reactions were performed using 0.1 mmol of 3-phenylcyclobutanone, 0.35 mmol of zinc, 2.0 mmol of H_2_O in 1.0 ml of a mixture of acetonitrile, toluene, and ethyl acetate (8 : 4 : 1) in the presence of 5 mol% of the catalyst under 1 atm of O_2_ at 35 °C.

^*b*^Determined by NMR analysis using dodecane as an internal standard.

With the appropriate conditions in hand, we then carried out the Baeyer–Villiger oxidation of 3-phenylcyclobutanone catalyzed by **Fl-Pep5-b** in the presence of an equimolar amount of other reactive substrate. Cyclooctene as a competitor remained intact during the desired conversion of the ketone (eqn (1)), whereas the preferential formation of cyclooctene oxide has occurred under mCPBA-based conditions (eqn (2)). Such excellent chemoselectivity was also observed in a competitive oxygenation of the ketone and thioanisole (eqn (3) and (4)). These results strongly suggest that peracid does not participate in the **Fl-Pep5** systems (eqn (1) and (3)) and, given that the ketone underwent oxidation predominantly, the corresponding **Fl_OOH_-Pep5** can be rather nucleophilic as opposed to the above chemoselective sulfoxidation.^18^1
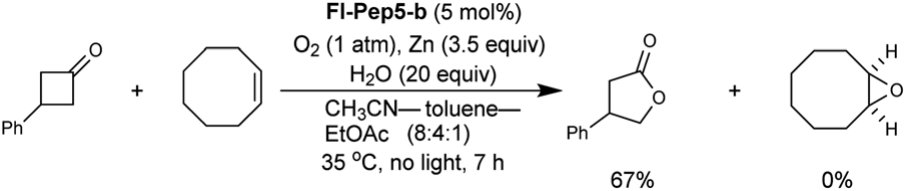

2
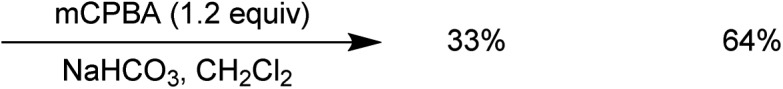

3
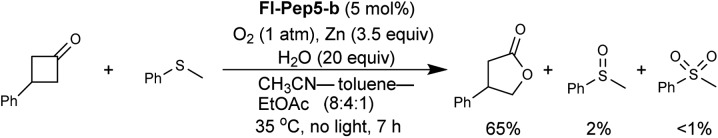

4
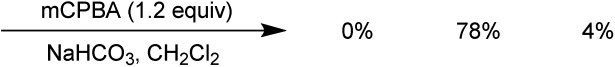



### Conformational analysis of **Fl-Pep**

We synthesized the soluble analogue of **Fl-Pep5**, 3-FlC2-Pro-Tyr-Asp-Ado-NH_2_, using Rink amide Resin to gain its conformational information by NMR spectroscopy (see ESI†). 3-FlC2-Pro-Tyr-Asp-Ado-NH_2_ was soluble in polar solvents such as dimethyl sulfoxide and methanol, but unfortunately, hardly soluble in less polar solvents such as acetonitrile, acetone, and chloroform. Thus, DMSO-*d*_6_ was inevitably used as the solvent, although it is quite unlike the actual reaction microenvironment that must be much less polar because of the hydrophobic nature of polystyrene resin. The NMR analysis showed that 3-FlC2-Pro-Tyr-Asp-Ado-NH_2_ forms two different conformers in DMSO-*d*_6_ at 25 °C in a ratio of ∼1.4 : 1, in which the major conformer has 3-FlC2-Pro amide bond in the *trans* conformation (58%), while that of the minor conformer is *cis* (42%). It should be noted that the observed *trans*–*cis* ratio for 3-FlC2-Pro-Tyr-Asp-Ado-NH_2_ in DMSO-*d*_6_ is similar to that for *N*-acetyl-l-proline *N*′-methylamide (Ac-Pro-NHMe, 65% *trans*)^19^ in the same solvent regardless of their large difference in structure and functionality. Given that Ac-Pro-NHMe predominantly favours the *trans*-C_7_ form (γ-turn) over other forms including the *cis* form in the gas phase and non-polar solvents,^20^ it is plausible that the flavopeptide moiety surrounded by the strongly hydrophobic environment in **Fl-Pep5** also populates the γ-turn form as included in the predicted stable conformation of 3-FlC2_4a(*R*)OOH_-Pro-Tyr-Asp-NHMe (Fig. 4). In fact, the catalytic activity of 3-FlC2-Pro-Tyr-Asp-Ado-NH_2_ was found to be much lower (7% yield in 24 h, see ESI†) than that of **Fl-Pep5-b** (Table 1, entry 17) in the sulfoxidation of thioanisole under the same conditions, showing the importance of the hydrophobic support resin that would make the flavopeptide conformationally profitable.^21^

### Mechanistic aspects of **Fl-Pep**-catalyzed aerobic oxygenations

**Fig. 6:**
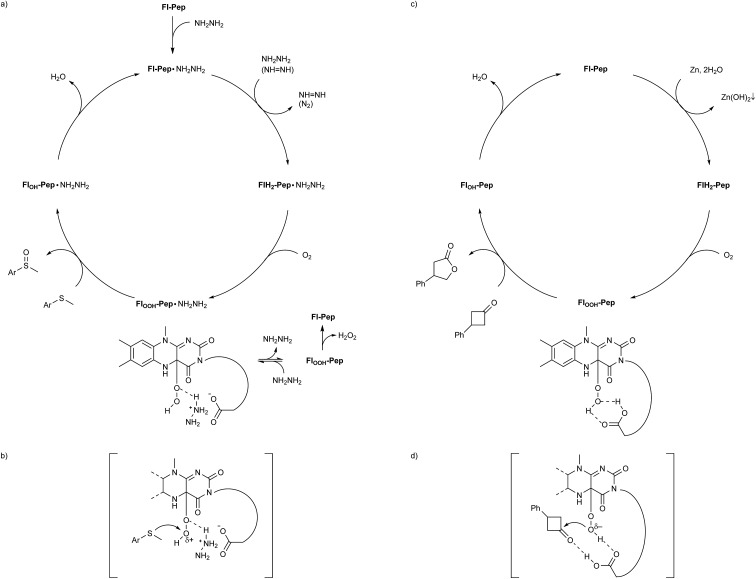
Proposed catalytic cycles and transition state models for **Fl-Pep**-catalyzed aerobic (a and b) sulfoxidation and (c and d) Baeyer–Villiger oxidation.

Given all the above experimental facts, it is plausible to consider that both the sulfoxidation and the Baeyer–Villiger oxidation catalyzed by **Fl-Pep** occur *via***Fl-Enz**-like mechanism (Fig. 6). As for the sulfoxidation, since effective **Fl-Pep1** and **Fl-Pep5** possess a carboxyl group that can readily react with an equivalent of NH_2_NH_2_ to be the corresponding salt (**Fl-Pep**·NH_2_NH_2_) *in situ*, the catalytic cycle (Fig. 6a) can be initiated by reducing **Fl-Pep**·NH_2_NH_2_ with another molecule of NH_2_NH_2_ to afford **FlH_2_-Pep**·NH_2_NH_2_ and diazene (NH = NH). The resulting NH = NH can also be used to reduce **Fl-Pep**·NH_2_NH_2_ from the second cycle by releasing N_2_. Molecular oxygen can be then inserted into the C(4a) of **FlH_2_-Pep**·NH_2_NH_2_ to give **Fl_OOH_-Pep**·NH_2_NH_2_. This hydroperoxy species may be effectively stabilized to perform subsequent monooxygenation of a substrate to give the corresponding 4a-hydroxy adduct (**Fl_OH_-Pep**·NH_2_NH_2_), which finally undergoes dehydration to regenerate **Fl-Pep**·NH_2_NH_2_. The Hammett study (Fig. 5) shows that the oxygen transfer from **Fl_OOH_-Pep**·NH_2_NH_2_ to a substrate is a rate-determining step of the proposed catalysis and takes place electrophilically. Although it is not trivial to verify the generation of the **Fl_OOH_-Pep** species spectroscopically due to the insolubility of resin, for the present, it is reasonable to understand that **Fl_OOH_-Pep** can be stabilized by means of intramolecular hydrogen bonds similar to those predicted by the DFT calculations (Fig. 2 and 4) including a probable coordination between C(4a)O of 3-FlC2 and ^+^NH_3_NH_2_ to make the hydroperoxy moiety electrophilic (Fig. 6b). The fact that Asp instead of Glu in AA3 enhanced the catalytic activity (Table 1, entries 2 *vs.* 10 and entries 11 *vs.* 17) could be rationalized by assuming such stabilization model that allows for a spatially less-forced intervention of NH_2_NH_2_ in between the carboxyl group and the hydroperoxy group. We consider that the presence of ^+^NH_3_NH_2_ is a key for stabilizing **Fl_OOH_-Pep**, which is a similar situation to **Fl_OOH_-Enz** that can be stabilized by complexation with NAD(P)^+^.5*c* Actually, an additional experiment on the effect of equivalents of hydrazine monohydrate for the present sulfoxidation revealed that the larger equivalents of NH_2_NH_2_, the faster reaction rate (see ESI†).^22^

On the other hand, **Fl_OOH_-Pep** in the Baeyer–Villiger oxidation can be formed *via* the reduction of **Fl-Pep** with Zn and H_2_O into **FlH_2_-Pep** followed by the oxygen insertion to **FlH_2_-Pep** and stabilized by computationally predicted hydrogen bonds (Fig. 4) including a cyclic coordination between 4aOOH of 3-FlC2 and COOH in the side chain of Asp, which then selectively oxidizes the ketone into the lactone to give **Fl_OH_-Pep** that finally release H_2_O to regenerate **Fl-Pep** (Fig. 6c). The nucleophilic activity of **Fl_OOH_-Pep** can be explained by assuming a transition state model involving simultaneous activation of the hydroperoxy moiety and the keto-carbonyl moiety by the COOH group (Fig. 6d), although the involvement of Zn^+^(OH) instead of H^+^ cannot be excluded for the moment.

## Conclusion

In conclusion, the first **Fl-Enz**-mimetic aerobic oxygenation reactions catalyzed by **Fl** under non-enzymatic conditions were realized. We predicted the structure of **Fl-Pep** that could stabilize the corresponding **Fl_OOH_-Pep** by a computational method, and synthesized the most promising **Fl-Pep1** and its analogies **Fl-Pep2–Fl-Pep5** as resin-immobilized peptides. Exploring their catalytic activity for aerobic sulfoxidation using hydrazine monohydrate as terminal reductant revealed that the computational design of **Fl-Pep** catalyst was reasonable although the fine-tuned **Fl-Pep5** showed superior activity than the original **Fl-Pep1**. On the other hand, the use of zinc as an alternative reductant under suitable conditions was found to allow for **Fl-Pep5**-catalyzed aerobic Baeyer–Villiger oxidation with excellent chemoselectivity. Multiple control experiment as well as mechanistic experiment suggested that both types of oxygenations could proceed *via***Fl-Enz**-like mechanism and the active species could be **Fl_OOH_** that had been efficiently used only in **Fl-Enz** so far. It is noteworthy that the electronic properties of the hydroperoxy moiety in **Fl_OOH_-Pep** can be orthogonally controlled by reductants and reaction conditions, realizing electrophilic sulfoxidation as well as nucleophilic Baeyer–Villiger oxidation in a highly chemoselective manner.^18^ We believe that the results are so important for the research fields of both flavin chemistry and peptide chemistry, because they not only provide new possibilities for the development of flavin catalysts as well as the fundamental study on flavin-containing monooxygenase but also demonstrate great potential of computational chemistry for the rational design of peptide-based catalysts.

## Supplementary Material

Supplementary information
