# Supplementary material for: Design of peptide-containing N5-unmodified neutral flavins that catalyze aerobic oxygenations
Source: Chem Sci. 2017 May 30;8(8):5468–75. doi: 10.1039/c7sc01933e (PMC6102831; doi:10.1039/c7sc01933e)
Supplement: Supplementary file 1 [file SC-008-C7SC01933E-s001.pdf]

Electronic Supplementary Material (ESI) for Chemical Science.
This journal is © The Royal Society of Chemistry 2017

Supporting Information

Design of Peptide-Containing *N*5-Unmodified Neutral Flavins That Catalyze Aerobic Oxygenations

Yukihiro Arakawa, Ken Yamanomoto, Hazuki Kita, Keiji Minagawa, Masami Tanaka, Naoki Haraguchi, Shinichi Itsuno and Yasushi Imada*

Table of Contents

1. **General Information** **S2**
2. **DFT conformational studies of flavopeptide (Fl-Pep)** **S2**
3. **Preparation of 3-FlC2-NH-PS** **S9**
4. **Preparation of Fl-Peps** **S9**
   - **4a. Fl-Pep1-a**, **Fl-Pep2-a**, **Fl-Pep3-a**, **Fl-Pep4-a**, and **Fl-Pep5-a**
   - **4b. Fl-Pep1-b**, **Fl-Pep2-b**, **Fl-Pep3-b**, **Fl-Pep4-b**, and **Fl-Pep5-b**
5. **Preparation of 3-FlC2-Pro-Tyr-Asp-Ado-NH$_2$** **S10**
6. **Aerobic oxidation of thioanisole with flavin catalyst** **S17**
   - **6a. Typical procedure**
   - **6b. Comparison under different conditions**
   - **6c. Product isolation**
   - **6d. Competitive reaction of *p*-substituted methyl phenyl sulfides for Fl-Pep1-a-catalyzed aerobic oxygenation**
7. **Aerobic Baeyer-Villiger oxidation of 3-phenylcyclobutan-1-one with flavin catalyst** **S19**
   - **7a. Typical procedure**
   - **7b. Comparison under different conditions**
   - **7c. Product isolation**
   - **7d. Competitive reaction**
8. **NMR spectra for 3-FlC2-Pro-Tyr-Asp-Ado-NH$_2$** **S26**
9. **References** **S29**

1. General information

NMR spectra were recorded using JEOL JNM-ECX-400 (^{1}H, 400 MHz), JNM-ECA-400 (^{1}H, 400 MHz), and JNM-ECA-500W (^{1}H, 500 MHz) spectrometers. Chemical shifts are reported in ppm using TMS or the residual solvent peak as a reference. Elemental analyses were carried out on a J-Science Lab JM10 micro corder. GC analyses were carried out on a Shimazu GC-2010 by using a DB-1 glass capillary column (0.25 mm×30 m). 3-Methyllumiflavin,[1] lumiflavin-3-acetic acid,[1,2] and Boc-Ado-OH[3] were prepared according to the literature procedures. Solid phase peptide syntheses were performed either on Intavis MultiPep CF automatically or under manual operation using (aminomethyl)polystyrene (70–90 mesh, 1% cross-linked, the N loadings were determined by elemental analysis in every lot: 1.21 mmol g^{-1} for the synthesis of **Fl-Pep1-a**, **Fl-Pep2-a**, **Fl-Pep3-a**, **Fl-Pep4-a**, and **Fl-Pep5-a**, 1.38 mmol g^{-1} for the synthesis of **Fl-Pep1-b**, **Fl-Pep2-b**, **Fl-Pep3-b**, **Fl-Pep4-b**, and **Fl-Pep5-b**, 1.48 mmol g^{-1} for the synthesis of 3-FlC2-NH-PS) purchased from Sigma-Aldrich or Rink amide Resin (100–200 mesh, 1% cross-linked, 0.53 mmol g^{-1} for the synthesis of 3-FlC2-Pro-Tyr-Asp-Ado-NH$_2$) purchased from Watanabe Chemical Industries, LTD. Fmoc-βAla-NH-PS and Boc-Ado-NH-PS were prepared according to the general procedure for peptide coupling described below. 3-Phenylcyclobutanone was prepared according to the reported procedure.[4] All other reagents were purchased from commercial supplies and used without purification.

2. DFT conformational studies of Fl-Pep

Spartan ’14 (Wavefunction, Inc.; Irvine, California, USA) was used to estimate stable conformations of 3-FlC2$_{4a(R)OOH}$-Pro-Glu-NHMe, 3-FlC2$_{4a(R)OOH}$-Pro-Tyr-NHMe, 3-FlC2$_{4a(R)OOH}$-Pro-Gly-NHMe, 3-FlC2$_{4a(R)OOH}$-Pro-Asp-Glu-NHMe, 3-FlC2$_{4a(R)OOH}$-Pro-Tyr-Glu-NHMe, 3-FlC2$_{4a(R)OOH}$-Pro-Tyr-Ser-NHMe, 3-FlC2$_{4a(R)OOH}$-Pro-Phe-Glu-NHMe, 3-FlC2$_{4a(S)OOH}$-Pro- Tyr-Glu-NHMe, 3-FlC2$_{4a(R)OOH}$-Pro-Tyr-Gln-NHMe, 3-FlC2$_{4a(R)OOH}$-βAla-Tyr-Glu-NHMe, and FlC2$_{4a(R)OOH}$-Pro-Tyr-Asp-NHMe. Monte Carlo conformational searches for these **Fl$_{OOH}$-Pep**s in MMFF were initially conducted. Among the resulting conformers, those with relative potential energy less than 15 kJ mol^{-1} and over Boltzmann distribution value of 0.02 were extracted and recalculated in DFT at B3LYP/6-31G* level. We analyzed the resulting lowest energy structures of **Fl$_{OOH}$-Pep**s to find promising hydrogen bonds (see the main manuscript) only in 3-FlC2$_{4a(R)OOH}$-Pro-Tyr-Glu-NHMe and FlC2$_{4a(R)OOH}$-Pro-Tyr-Asp-NHMe.

Conformers within 10 kJ mol^{-1} are shown below, where their relative potential energy values in kJ mol^{-1} are given in parentheses and hydrogen bonds are given in blue dotted line.

- 3-FlC2$_{4a(R)OOH}$-Pro-Tyr-Glu-NHMe

M0004(0.00)

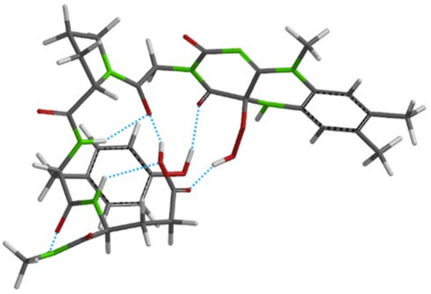

M0004 (side)

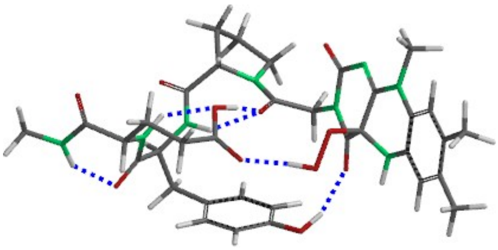

M0008 (1.09)

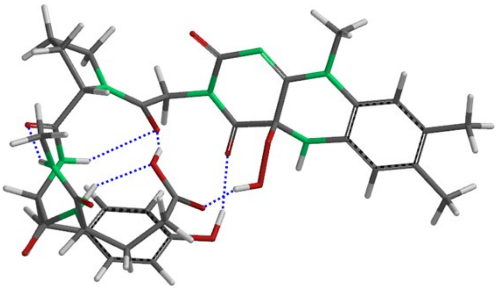

M0001 (1.16)

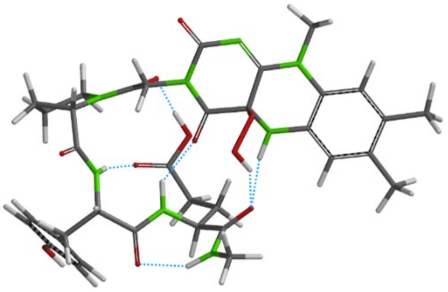

M0003 (1.40)

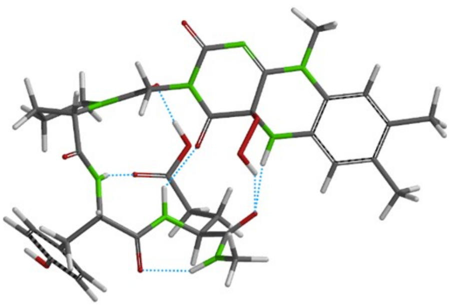

M0011 (2.08)

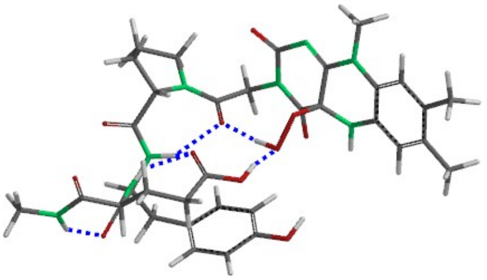

M0002 (2.97)

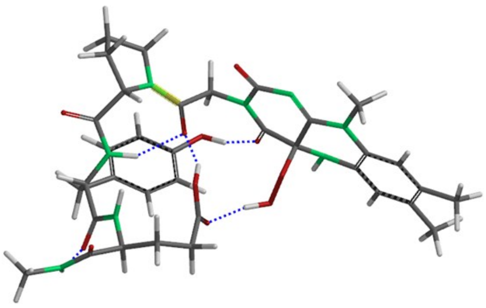

M0005 (4.62)

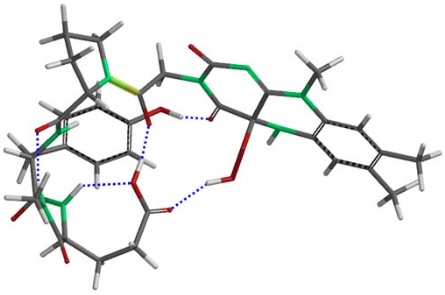

- 3-FlC2$_{4a(R)OOH}$-Pro-Phe-Glu-NHMe

M0004 (0.00)

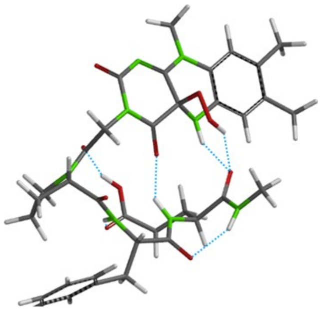

M0001 (0.59)

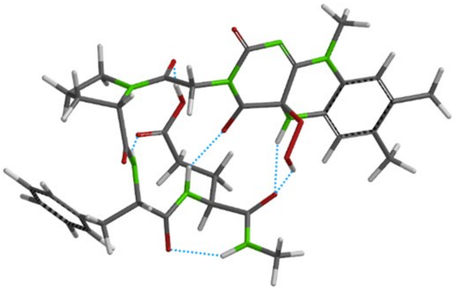

M0002 (0.59)

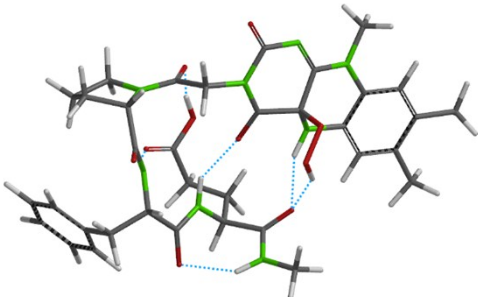

- 3-FlC2$_{4a(R)OOH}$-Pro-Asp-Glu-NHMe

M0002 (0.00)

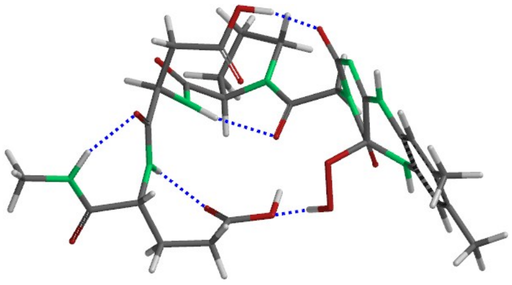

M0004 (3.55)

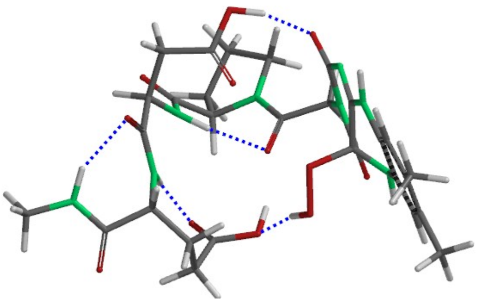

M0001 (19.74)

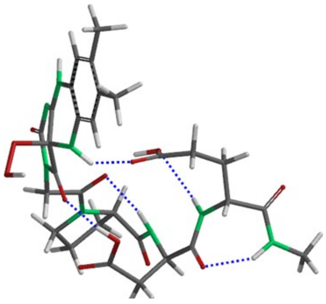

- 3-FlC2$_{4a(R)OOH}$-Pro-Tyr-Ser-NHMe

M0001 (0.00)

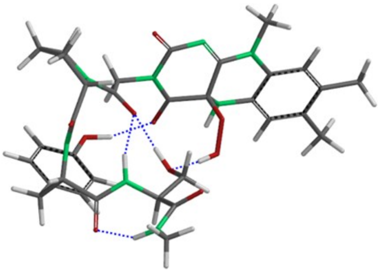

M0002 (0.22)

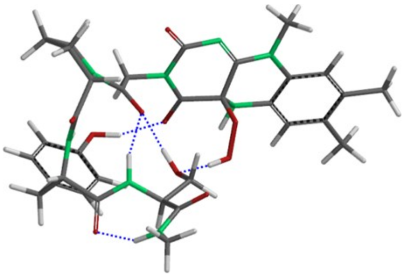

M0008 (5.83)

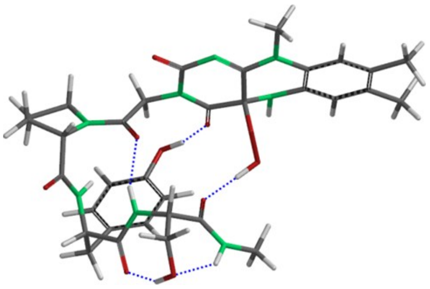

- 3-FlC2$_{4a(\mathit{S})OOH}$-Pro-Tyr-Glu-NHMe

M0002 (0.00)

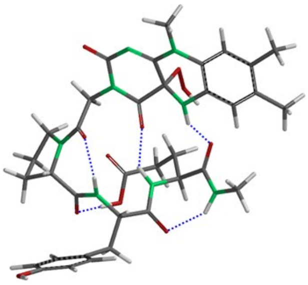

M0003 (0.30)

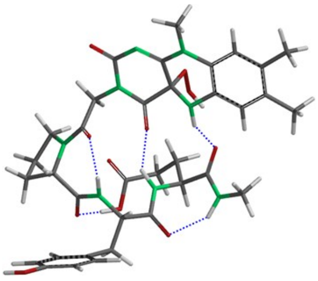

M0001 (18.79)

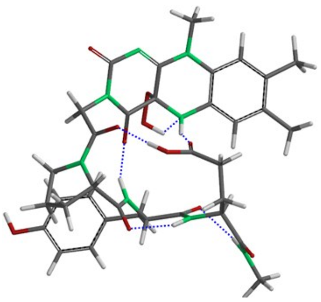

- 3-FlC2$_{4a(R)OOH}$-Pro-Glu-NHMe

M0010 (0.00)

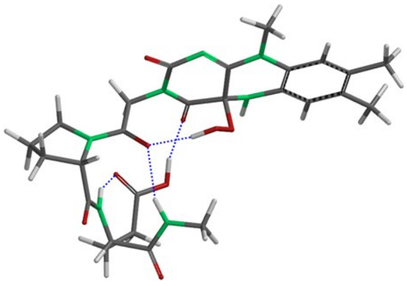

M0006 (3.08)

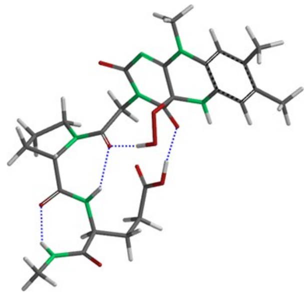

M0005 (4.53)

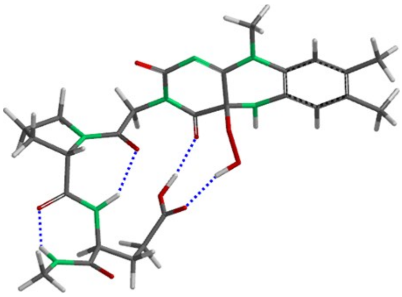

M0009 (8.78)

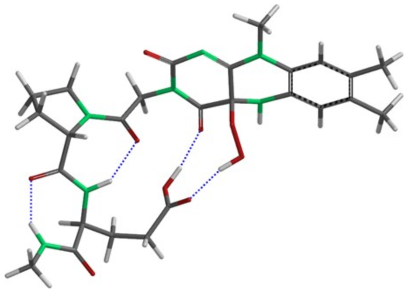

- 3-FlC2$_{4a(R)OOH}$-Pro-Tyr-NHMe

M0006 (0.00)

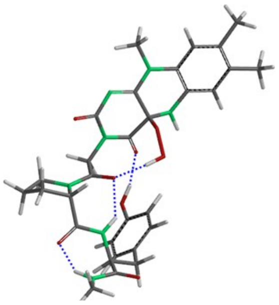

M0002 (11.44)

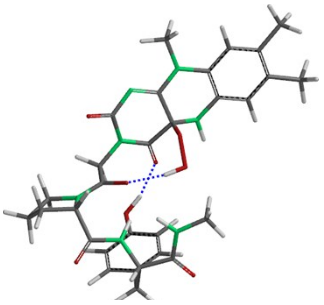

- 3-FlC2$_{4a(R)OOH}$-Pro-Gly-NHMe

M0004 (0.00)

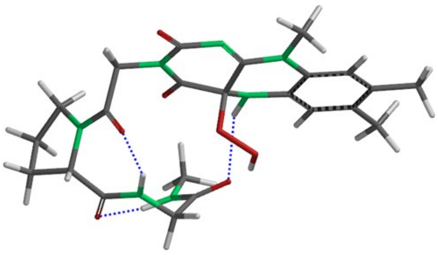

M0003 (3.56)

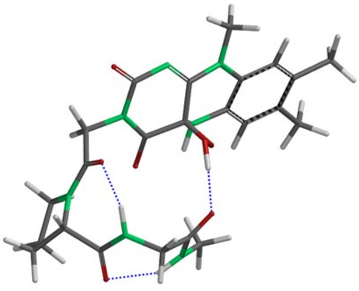

M0001 (3.57)

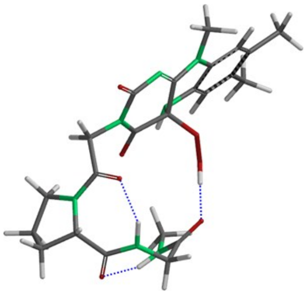

M0002 (5.79)

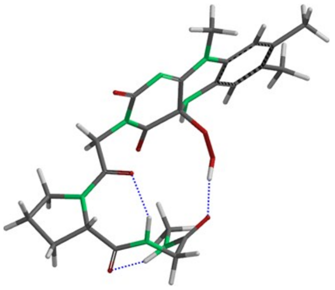

- 3-FlC2$_{4a(R)OOH}$-Pro-Tyr-Gln-NHMe

M0003 (0.00)

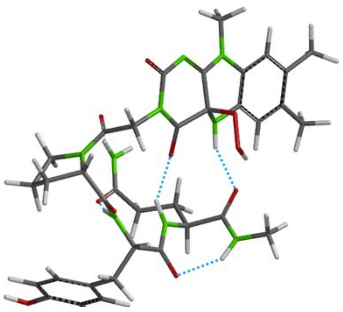

M0002 (0.09)

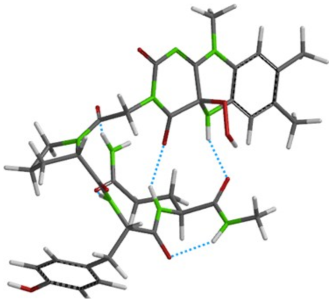

M0001 (0.19)

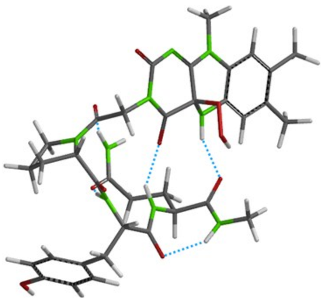

M0007 (4.92)

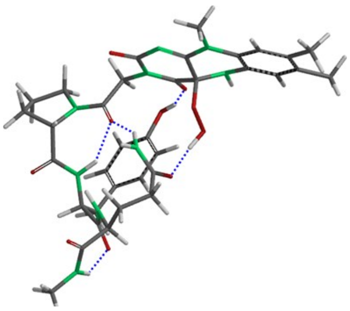

- 3-FlC2$_{4a(R)OOH}$ -βAla-Tyr-Glu-NHMe

M0002 (0.00)

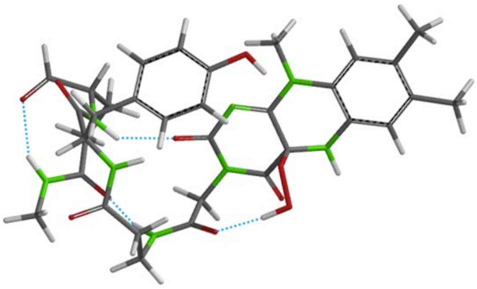

M0001 (17.67)

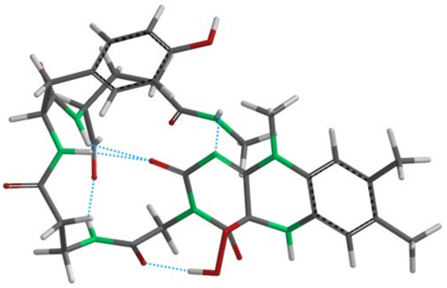

- 3-FlC2$_{4a(R)OOH}$-Pro-Tyr-Asp-NHMe

M0014 (0.00)

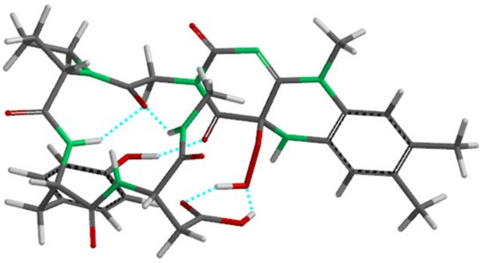

M0012 (0.10)

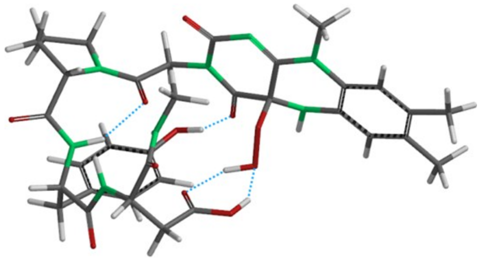

M0002 (5.83)

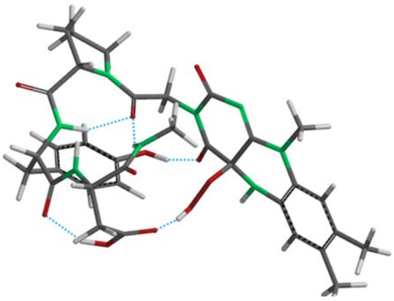

M0009 (6.79)

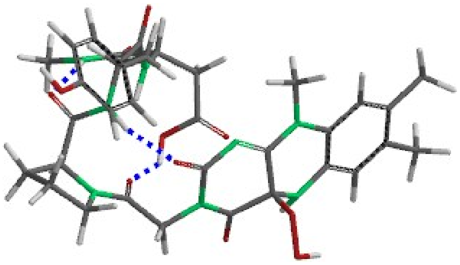

3. Preparation of 3-FlC2-NH-PS

//To (aminomethyl)polystyrene pre-swollen in DMF was added a solution of lumiflavin-3-acetic acid (2.5 equiv), HCTU (2.5 equiv) and *N*-ethyldiisopropylamine (7.5 equiv), and the mixture was agitated for 2 h at room temperature. The coupling reaction was monitored by qualitative Kaiser Test.[5] The suspension was washed with DMF repeatedly until the solution layer becomes colorless and then with CH_2Cl_2 (3×), and the resulting resin was dried in *vacuo* at room temperature to give 3-FlC2-NH-PS. The catalyst loading of 3-FlC2-NH-PS was determined to be 1.00 mmol g^{-1} by elemental analysis. Found: N 6.99%, H 6.82%, C 77.47%.

4. Preparation of flavopeptides (Fl-Peps)

4a. Fl-Pep1-a, Fl-Pep2-a, Fl-Pep3-a, Fl-Pep4-a, and Fl-Pep5-a

These flavopeptides were prepared through the automated synthesis of Fmoc-AA1-AA2-AA3-βAla-NH-PS (where AA1=Pro or βAla, AA2=Tyr(*t*-Bu) or Phe, AA3=Glu(O*t*-Bu), Asp(O*t*-Bu), or Gln(Trt)) from Fmoc-βAla-NH-PS followed by the manual coupling of lumiflavin-3-acetic acid after Fmoc-deprotection, and finally *t*-Bu or Trt-deprotection. The automated processes were carried out using Fmoc-βAla-NH-PS (215 mg, 245 μmol), Fmoc-amino acids (0.5 M in DMF, 5.25 equiv), HBTU (0.5 M in DMF, 5.25 equiv) as a coupling agent, *N*-methylmorpholine (3.9 M in DMF, 9.60 equiv) as a base, Ac_2O (0.54 M in DMF, 13.2 equiv) as a capping agent, and DMF or NMP (when Fmoc-Pro-OH was used) as solvents under conditions recommended by Intavis. Fmoc-deprotection, the coupling of lumiflavin-3-acetic acid, and *t*-Bu or Trt deprotection under manual operation were carried out following the general procedures described below. Catalyst loadings of **Fl-Pep1-a**, **Fl-Pep2-a**, **Fl-Pep3-a**, **Fl-Pep4-a**, and **Fl-Pep5-a** were determined to be 0.48, 0.42, 0.47, 0.53, and 0.48 mmol g^{-1}, respectively, by quantitative Fmoc test of Fmoc-AA1-AA2-AA3-βAla-NH-PS.

General procedure for Fmoc-deprotection

A 20% v/v solution of piperidine in DMF was added to the Fmoc-protected resin pre-swollen in DMF and the reaction mixture was agitated for 10 min. The solution phase was drained and the resin was again treated with 20% v/v solution of piperidine for another 15 min. The resin was then washed with DMF (3×), DMF/CH_2Cl_2 (4:1) (5×), and CH_2Cl_2 (3×).

General procedure for the coupling of lumiflavin-3-acetic acid

To the resin modified by *N*-terminus unprotected peptide pre-swollen in DMF was added a solution of lumiflavin-3-acetic acid (2.5 equiv), HCTU (2.5 equiv), and *N*-ethyldiisopropylamine (7.5 equiv), and the mixture was agitated for 2 h. The suspension was washed with DMF repeatedly until the solution layer becomes colorless, then with DMF/CH_2Cl_2 (4:1) (5×), and with CH_2Cl_2 (3×).

The coupling reaction was monitored by either qualitative Kaiser test[5] (**Fl-Pep2-a**) or chloranil test[6] (**Fl-Pep1-a**, **Fl-Pep3-a**, **Fl-Pep4-a**, and **Fl-Pep5-a**).

General procedure for *t*-Bu or Trt-deprotection

After the coupling of lumiflavin-3-acetic acid, a mixture of TFA/CH_2Cl_2 (2:1) was added to the resulting resin pre-swollen in CH_2Cl_2, and the reaction mixture was agitated for 1 h. The solution phase was drained and the resin was again treated with a mixture of TFA/CH_2Cl_2 (2:1) for 20 min. Finally, the resin was washed with CH_2Cl_2 (6×) and dried in *vacuo* to afford **Fl-Pep1-a**–**Fl-Pep5-a**.

4b. Fl-Pep1-b, **Fl-Pep2-b**, **Fl-Pep3-b**, **Fl-Pep4-b**, and **Fl-Pep5-b**

These flavopeptides were prepared by manual solid-phase peptide synthesis from Boc-Ado-NH-PS following the general procedure for peptide coupling described below along with the above general procedures for Fmoc-deprotection, the coupling of lumiflavin-3-acetic acid, and *t*-Bu or Trt-deprotection. Boc-Ado-NH-PS was treated with a mixture of TFA/CH_2Cl_2 (2:1) twice (the 1st time: 1 h, the 2nd time: 20 min) to remove the Boc group and then washed with CH_2Cl_2 (3×), 5% v/v *N*-ethyldiisopropylamine in CH_2Cl_2 (3×), and CH_2Cl_2 (6×) prior to use. Catalyst loadings of **Fl-Pep1-b**, **Fl-Pep2-b**, **Fl-Pep3-b**, **Fl-Pep4-b**, and **Fl-Pep5-b** were determined to be 0.54, 0.51, 0.62, 0.59, and 0.55 mmol g^{-1}, respectively, by quantitative Fmoc test of Fmoc-AA1-AA2-AA3- Ado-NH-PS.

General procedure for peptide coupling

To the amino functionalized resin pre-swollen in DMF was added a solution of the Fmoc-amino acid (2.5 equiv), HCTU (2.5 equiv), and *N*-ethyldiisopropylamine (7.5 equiv), and the mixture was agitated for 1.5 h. The suspension was washed with DMF (5×), DMF/CH_2Cl_2 (4:1) (5×), and CH_2Cl_2 (3×). The coupling reaction was monitored by qualitative Kaiser[5] and chloranil tests[6] (secondary amine).

5. Preparation of 3-FlC2-Pro-Tyr-Asp-Ado-NH_2

3-FlC2-Pro-Tyr-Asp-Ado-NH_2 was prepared on Rink amide Resin following the general protocol for Fmoc solid phase peptide synthesis. After the flavopeptide sequence was constructed, a mixture of TFA/CH_2Cl_2 (2:1) was added to the modified resin pre-swollen in CH_2Cl_2, and the reaction mixture was agitated for 1 h for *t*-Bu and Trt-deprotection and cleavage of the flavopeptide. The solution phase was pooled and the resin was again treated with a mixture of TFA/CH_2Cl_2 (2:1) for 20 min. All volatiles were removed from the combined filtrates under reduced pressure. Et_2O was added to the residue, and the resulting precipitate was washed with Et_2O and recrystallized from

a mixture of methanol and ethanol to afford the flavopeptide as yellow solid, which was characterized by NMR (^{1}H, ^{13}C, COSY, HMBC, HMQC, ROESY) and mass spectroscopy (MALDI-TOF). ^{1}H-NMR (600 MHz, DMSO-d_6, δ): **Major conformer (Mj)** : 1.19 (14H, AdoH$^{\gamma-\iota}$), 1.28 (2H, AdoH$^{\kappa}$) 1.44 (2H, AdoH$^{\beta}$), 1.68–1.79 (m, 2H, ProH$^{\beta}$, ProH$^{\gamma}$), 1.87–1.90 (m, 1H, ProH$^{\gamma'}$), 1.96 (m,1H, ProH$^{\beta'}$), 2.00 (t, J = 7.4 Hz, 2H, AdoH$^{\alpha}$), 2.37 (dd, J = 6.9, 16.3 Hz, 1H, AspH$^{\beta}$), 2.40 (s, 3H, Fl7-CH_3), 2.51 (s, 3H, Fl8-CH_3), 2.56 (dd, J = 6.9, 16.4 Hz, 1H, AspH$^{\beta'}$), 2.72 (dd, J = 9.5, 13.7 Hz, 1H, TyrH$^{\beta}$), 2.89–2.92 (3H, TyrH$^{\beta'}$, AdoH$^{\lambda}$), 3.61–3.71 (m, 2H, ProH$^{\delta,\delta'}$), 4.00 (s, 3H, Fl10-CH_3), 4.24–4.28 (m, 2H, ProH$^{\alpha}$, TyrH$^{\alpha}$), 4.38 (ddd, J = 6.9, 6.9, 8.1 Hz, 1H, AspH$^{\alpha}$), 4.72 (d, J = 15.8 Hz, 1H, Fl3-CH_2), 4.78 (d, J = 15.9 Hz, 1H, Fl3-CH_2'), 6.62 (d, J = 8.5 Hz, 2H, TyrArH^{ortho}), 6.65 (br, 1H, CONH_2), 7.02 (d, J = 8.5 Hz, 2H, TyrArH^{meta}), 7.23 (t, J = 5.6 Hz, 1H, AdoNH), 7.78 (d, J = 8.1 Hz, 1H, AspNH), 7.81 (d, J = 7.7 Hz, 1H, TyrNH), 7.83 (1H, Fl9-H), 7.94 (1H, Fl6-H); **Minor conformer (Mn)** : 1.19 (14H, AdoH$^{\gamma-\iota}$), 1.34 (2H, AdoH$^{\kappa}$), 1.44 (2H, AdoH$^{\beta}$), 1.68–1.79 (m, 2H, ProH$^{\gamma,\gamma'}$), 1.87–1.90 (m, 1H, ProH$^{\beta}$), 1.99 (t, J = 7.1 Hz, 2H, AdoH$^{\alpha}$), 2.23 (ddd, J = 8.5, 12.8, 17.3 Hz, 1H, ProH$^{\beta'}$), 2.41 (s, 3H, Fl7-CH_3), 2.51 (s, 3H, Fl8-CH_3), 2.54 (dd, J = 6.7, 16.5 Hz, 1H, AspH$^{\beta}$), 2.64 (dd, J = 10.7, 13.8 Hz, 1H, TyrH$^{\beta}$), 2.65 (dd, J = 6.0, 16.5 Hz, 1H, AspH$^{\beta'}$), 2.95 (dd, J = 4.3, 13.7 Hz, 1H, TyrH$^{\beta'}$), 2.99–3.01 (m, 2H, AdoH$^{\lambda}$), 3.28–3.38 (m, 2H, ProH$^{\delta,\delta'}$), 3.93 (s, 2H, Fl3-CH_2), 4.01 (s, 3H, Fl10-CH_3), 4.43 (dd, J = 3.6, 8.5 Hz, 1H, ProH$^{\alpha}$), 4.49 (ddd, J = 6.5, 6.5, 7.4 Hz, 1H, AspH$^{\alpha}$), 4.53 (ddd, J = 4.7, 8.5, 10.7 Hz, 1H, TyrH$^{\alpha}$), 6.44 (d, J = 8.4 Hz, 2H, TyrArH^{ortho}), 7.02 (d, J = 8.5 Hz, 2H, TyrArH^{meta}), 7.19 (br, 1H, CONH_2), 7.57 (t, J = 5.6 Hz, 1H, AdoNH), 7.83 (1H, Fl9-H), 7.94 (1H, Fl6-H), 8.21 (d, J = 7.7 Hz, 1H, AspNH), 8.40 (d, J = 8.4 Hz, 1H, TyrNH); ^{13}C-NMR (151 MHz, DMSO-d_6, δ): 18.7 (Fl7-*C*H$_3$, **Mj** and **Mn**), 20.6 (Fl8-*C*H$_3$, **Mj** and **Mn**), 22.2 (ProC$^{\gamma}$, **Mn**), 24.1 (ProC$^{\gamma}$, **Mj**), 25.1 (AdoC$^{\beta}$, **Mj** and **Mn**), 25.3, 26.1, 26.2, 26.3, 29.1/29.0/28.9/28.8/28.7 (including ProC$^{\beta}$, **Mj**), 31.9/31.8 (Fl10-*C*H$_3$, **Mj** and **Mn**; ProC$^{\beta}$, **Mn**), 35.1 (AdoC$^{\alpha}$, **Mj** and **Mn**), 35.4, 35.9, 36.0, 36.2, 36.5, 38.6/38.3 (AdoC$^{\lambda}$, **Mj** and **Mn**), 42.2 (Fl3-*C*H$_2$, **Mn**), 43.0 (Fl3-*C*H$_2$, **Mj**), 46.3 (ProC$^{\delta}$, **Mj**), 46.9 (ProC$^{\delta}$, **Mn**), 49.5 (AspC$^{\alpha}$, **Mj** and **Mn**), 54.3 (TyrC$^{\alpha}$, **Mn**), 54.8 (TyrC$^{\alpha}$, **Mj**), 58.8 (ProC$^{\alpha}$, **Mn**), 60.0 (ProC$^{\alpha}$, **Mj**), 114.7 (TyrArC^{ortho}), 114.9 (TyrArC^{ortho}), 116.5/116.4 (FlC^{9}, **Mj** and **Mn**), 127.5, 127.7, 129.9 (TyrArC^{meta}, **Mn**), 130.0 (TyrArC^{meta}, **Mj**), 130.9 (FlC^{6}, **Mj** and **Mn**), 131.7 (FlC^{9a}), 134.1/134.0 (FlC^{5a}), 136.2/136.1/135.8/135.6 (FlC^{7}/FlC^{4a}), 147.3/147.0 (FlC^{8}), 149.0 (FlC^{10a}, **Mn**), 149.1 (FlC^{10a}, **Mj**), 154.2 (FlC^{4}, **Mn**), 154.5 (FlC^{4}, **Mj**), 155.6 (TyrArC^{ipso}, **Mn**), 155.8 (TyrArC^{ipso}, **Mj**), 158.7 (FlC^{2}, **Mn**), 159.3 (FlC^{2}, **Mj**), 165.5 (ProN*C*O, **Mn**), 166.2 (ProN*C*O, **Mj**), 169.7 (Asp*C*ONH, **Mj**), 169.8 (Asp*C*ONH, **Mn**), 171.7/171.6/171.4/171.1/171.0/170.8 (Tyr*C*ONH, Pro*C*ONH, AspC^{γ}O, **Mj** and **Mn**), 174.3 (*C*ONH$_2$, **Mj** and **Mn**); MS (MALDI-TOF): m/z calculated for $C_{45}H_{59}N_9O_{10}$ [M + Na]$^{+}$ 908.4283, found 908.4333.

COSY cross-peaks

F1 (ppm)	F2 (ppm)	Assignment	F1 (ppm)	F2 (ppm)	Assignment
1.19<br>1.44	1.44<br>1.19	AdoH$^{\gamma-\iota}$– AdoH$^{\beta}$<br>(**Mj** and **Mn**)	1.28<br>1.19	1.19<br>1.28	AdoH$^{\kappa}$–AdoH$^{\gamma-\iota}$ (**Mj**)
1.28<br>2.89–2.92	2.89–2.92<br>1.28	AdoH$^{\kappa}$–AdoH$^{\lambda}$ (**Mj**)	1.34<br>1.19	1.19<br>1.34	AdoH$^{\kappa}$–AdoH$^{\gamma-\iota}$ (**Mn**)
1.34<br>2.99–3.01	2.99–3.01<br>1.34	AdoH$^{\kappa}$–AdoH$^{\lambda}$ (**Mn**)	1.44<br>1.19	1.19<br>1.44	AdoH$^{\beta}$–AdoH$^{\gamma-\iota}$<br>(**Mj** and **Mn**)
1.44<br>2.00	2.00<br>1.44	AdoH$^{\beta}$–AdoH$^{\alpha}$ (**Mj**)	1.68–1.79<br>1.87–1.90	1.87–1.90<br>1.68–1.79	ProH$^{\beta}$ /ProH$^{\gamma}$–ProH$^{\gamma'}$(**Mj**)<br>ProH$^{\gamma/\gamma'}$–ProH$^{\beta}$ (**Mn**)
1.68–1.79<br>1.96	1.96<br>1.68–1.79	ProH$^{\beta}$ /ProH$^{\gamma}$–ProH$^{\beta'}$(**Mj**)	1.68–1.79<br>2.23	2.23<br>1.68–1.79	ProH$^{\gamma,\gamma'}$–ProH$^{\beta'}$ (**Mn**)
1.68–1.79<br>3.28–3.38	3.28–3.38<br>1.68–1.79	ProH$^{\gamma,\gamma'}$–ProH$^{\delta,\delta'}$ (**Mn**)	1.68–1.79<br>3.61–3.71	3.61–3.71<br>1.68–1.79	ProH$^{\gamma}$–ProH$^{\delta,\delta'}$ (**Mj**)
1.68–1.79<br>4.24–4.28	4.24–4.28<br>1.68–1.79	ProH$^{\gamma'}$–ProH$^{\alpha}$ (**Mj**)	1.87–1.90<br>1.96	1.96<br>1.87–1.90	ProH$^{\gamma'}$–ProH$^{\beta'}$ (**Mj**)
1.87–1.90<br>2.23	2.23<br>1.87–1.90	ProH$^{\beta}$–ProH$^{\beta'}$ (**Mn**)	1.87–1.90<br>3.61–3.71	3.61–3.71<br>1.87–1.90	ProH$^{\gamma,\gamma'}$–ProH$^{\delta,\delta'}$ (**Mj**)
1.87–1.90<br>4.43	4.43<br>1.87–1.90	ProH$^{\beta}$–ProH$^{\alpha}$ (**Mn**)	1.96<br>4.24–4.28	4.24–4.28<br>1.96	ProH$^{\beta'}$–ProH$^{\alpha}$ (**Mj**)
2.23<br>4.43	4.43<br>2.23	ProH$^{\beta'}$–ProH$^{\alpha}$ (**Mn**)	2.37<br>2.56	2.56<br>2.37	AspH$^{\beta}$–AspH$^{\beta'}$ (**Mj**)
2.37<br>4.38	4.38<br>2.37	AspH$^{\beta}$–AspH$^{\alpha}$ (**Mj**)	2.40<br>7.94	7.94<br>2.40	Fl7-CH_3–Fl6-H (**Mj**)
2.41<br>7.94	7.94<br>2.41	Fl7-CH_3–Fl6-H (**Mn**)	2.51<br>7.83	7.83<br>2.51	Fl8-CH_3–Fl9-H<br>(**Mj** and **Mn**)
2.54<br>4.49	4.49<br>2.54	AspH$^{\beta}$–AspH$^{\alpha}$ (**Mn**)	2.54<br>2.65	2.65<br>2.54	AspH$^{\beta}$–AspH$^{\beta'}$ (**Mn**)
2.56<br>4.38	4.38<br>2.56	AspH$^{\beta'}$–AspH$^{\alpha}$ (**Mj**)	2.64<br>2.95	2.95<br>2.64	TyrH$^{\beta}$–TyrH$^{\beta'}$ (**Mn**)
2.64<br>4.53	4.53<br>2.64	TyrH$^{\beta}$–TyrH$^{\alpha}$ (**Mn**)	2.72<br>2.89–2.92	2.89–2.92<br>2.72	TyrH$^{\beta}$–TyrH$^{\beta'}$ (**Mj**)
2.72<br>4.24–4.28	4.24–4.28<br>2.72	TyrH$^{\beta}$–TyrH$^{\alpha}$ (**Mj**)	2.89–2.92<br>4.24–4.28	4.24–4.28<br>2.89–2.92	TyrH$^{\beta'}$–TyrH$^{\alpha}$ (**Mj**)
2.89–2.92<br>7.23	7.23<br>2.89–2.92	AdoH$^{\lambda}$–AdoNH (**Mj**)	2.95<br>4.53	4.53<br>2.95	TyrH$^{\beta'}$–TyrH$^{\alpha}$ (**Mn**)
2.99–3.01<br>7.57	7.57<br>2.99–3.01	AdoH$^{\lambda}$–AdoNH (**Mn**)	4.24–4.28<br>7.81	7.81<br>4.24–4.28	TyrH$^{\alpha}$–TyrNH (**Mj**)
4.38<br>7.78	7.78<br>4.38	AspH$^{\alpha}$–AspNH (**Mj**)	4.49<br>2.65	2.65<br>4.49	AspH$^{\alpha}$–AspH$^{\beta'}$ (**Mn**)
4.49<br>8.21	8.21<br>4.49	AspH$^{\alpha}$–AspNH (**Mn**)	4.53<br>8.40	8.40<br>4.53	TyrH$^{\alpha}$–TyrNH (**Mn**)
6.44<br>7.02	7.02<br>6.44	TyrArH^{ortho}–TyrArH^{meta}<br>(**Mn**)	6.62<br>7.02	7.02<br>6.62	TyrArH^{ortho}–TyrArH^{meta}<br>(**Mj**)

HMQC cross-peaks

F1 (ppm)	F2 (ppm)	Assignment	F1 (ppm)	F2 (ppm)	Assignment
18.7	2.40/2.41	Fl7-*C*H_3– Fl7-C*H_3*<br>(**Mj** and **Mn**)	20.6	2.51	Fl8-*C*H_3–Fl8-C*H_3*<br>(**Mj** and **Mn**)
22.2	1.68–1.79	ProC$^{\gamma}$–ProH$^{\gamma,\gamma'}$ (**Mn**)	24.1	1.87–1.90	ProC$^{\gamma}$–ProH$^{\gamma'}$ (**Mj**)
25.1	1.44	AdoC$^{\beta}$–AdoH$^{\beta}$<br>(**Mj** and **Mn**)	26.1–26.3	1.19	–AdoH$^{\gamma-\iota}$<br>(**Mj** and **Mn**)
29.1–28.7	1.19	–AdoH$^{\gamma-\iota}$<br>(**Mj** and **Mn**)	29.1–28.7	1.28	AdoC$^{\kappa}$–AdoH$^{\kappa}$ (**Mj**)

HMQC cross-peaks (continued)

29.1–28.7	1.34	AdoC$^{\kappa}$–AdoH$^{\kappa}$ (**Mn**)	29.1–28.7	1.68–1.79	ProC$^{\beta}$–ProH$^{\beta}$ (**Mj**)
29.1–28.7	1.96	ProC$^{\beta}$–ProH$^{\beta'}$ (**Mj**)	31.9/31.8	1.87–1.90	ProC$^{\beta}$–ProH$^{\beta}$ (**Mn**)
31.9/31.8	2.23	ProC$^{\beta}$–ProH$^{\beta'}$ (**Mn**)	31.9/31.8	4.00/4.01	Fl10-CH$_3$–Fl10-CH_3 (**Mj** and **Mn**)
35.1	1.99/2.00	AdoC$^{\alpha}$–AdoH$^{\alpha}$ (**Mj** and **Mn**)	36.5–35.4	2.95	TyrC$^{\beta}$–TyrH$^{\beta'}$ (**Mn**)
36.5–35.4	2.89–2.92	TyrC$^{\beta}$–TyrH$^{\beta'}$ (**Mj**)	36.5–35.4	2.72	TyrC$^{\beta}$–TyrH$^{\beta}$ (**Mj**)
36.5–35.4	2.65	AspC$^{\beta}$–AspH$^{\beta'}$ (**Mn**)	36.5–35.4	2.64	TyrC$^{\beta}$–TyrH$^{\beta}$ (**Mn**)
36.5–35.4	2.56	AspC$^{\beta}$–AspH$^{\beta'}$ (**Mj**)	36.5–35.4	2.54	AspC$^{\beta}$–AspH$^{\beta}$ (**Mn**)
36.5–35.4	2.37	AspC$^{\beta}$–AspH$^{\beta}$ (**Mj**)	38.6/38.3	2.89–2.92	AdoC$^{\lambda}$–AdoH$^{\lambda}$ (**Mj**)
38.6/38.3	2.99–3.01	AdoC$^{\lambda}$–AdoH$^{\lambda}$ (**Mn**)	42.2	3.93	Fl3-CH$_2$–Fl3-CH_2 (**Mn**)
43.0	4.72	Fl3-CH$_2$–Fl3-CH_2 (**Mj**)	43.0	4.78	Fl3-CH$_2$–Fl3-CH_2’ (**Mj**)
46.3	3.61–3.71	ProC$^{\delta}$–ProH$^{\delta,\delta'}$ (**Mj**)	46.9	3.28–3.38	ProC$^{\delta}$–ProH$^{\delta,\delta'}$ (**Mn**)
49.5	4.38	AspC$^{\alpha}$–AspH$^{\alpha}$ (**Mj**)	49.5	4.49	AspC$^{\alpha}$–AspH$^{\alpha}$ (**Mn**)
54.3	4.53	TyrC$^{\alpha}$–TyrH$^{\alpha}$ (**Mn**)	54.8	4.24–4.28	TyrC$^{\alpha}$–TyrH$^{\alpha}$ (**Mj**)
58.8	4.43	ProC$^{\alpha}$–ProH$^{\alpha}$ (**Mn**)	60.0	4.24–4.28	ProC$^{\alpha}$–ProH$^{\alpha}$ (**Mj**)
114.7	6.44	TyrArC^{ortho}–TyrArH^{ortho} (**Mn**)	114.9	6.62	TyrArC^{ortho}–TyrArH^{ortho} (**Mj**)
116.5/116.4	7.83	FlC^{9}–Fl9-H (**Mj** and **Mn**)	129.9	7.02	TyrArC^{meta}–TyrArH^{meta} (**Mn**)
130.0	7.02	TyrArC^{meta}–TyrArH^{meta} (**Mj**)	130.9	7.94	FlC^{6}–Fl6-H (**Mj** and **Mn**)

HMBC cross-peaks

F1 (ppm)	F2 (ppm)	Assignment	F1 (ppm)	F2 (ppm)	Assignment
18.7	7.94	Fl7-CH$_3$–Fl6-H (**Mj** and **Mn**)	20.6	7.83	Fl8-CH$_3$–Fl9-H (**Mj** and **Mn**)
22.2	3.28–3.38	ProC$^{\gamma}$–ProH$^{\delta,\delta'}$ (**Mn**)	24.1	3.61–3.71	ProC$^{\gamma}$ ProH$^{\delta,\delta'}$ (**Mj**)
25.1	1.19	AdoC$^{\beta}$–AdoH$^{\gamma-\iota}$ (**Mj** and **Mn**)	25.1	1.99/2.00	AdoC$^{\beta}$–AdoH$^{\alpha}$ (**Mj** and **Mn**)
26.1–26.3	1.19	–AdoH$^{\gamma-\iota}$ (**Mj** and **Mn**)	26.1–26.3	1.28	–AdoH$^{\kappa}$ (**Mj**)
26.1–26.3	1.34	–AdoH$^{\kappa}$ (**Mn**)	26.1–26.3	2.89–2.92	–AdoH$^{\lambda}$ (**Mj**)
26.1–26.3	2.99–3.01	–AdoH$^{\lambda}$ (**Mn**)	26.1–26.3	1.19	–AdoH$^{\gamma-\iota}$ (**Mj** and **Mn**)
29.1–28.7	1.28	–AdoH$^{\kappa}$ (**Mj**)	29.1–28.7	1.34	–AdoH$^{\kappa}$ (**Mn**)
29.1–28.7	1.44	–AdoH$^{\beta}$ (**Mj** and **Mn**)	29.1–28.7	1.96	–ProH$^{\beta'}$ (**Mj**)
29.1–28.7	2.89–2.92	–AdoH$^{\lambda}$ (**Mj**)	29.1–28.7	2.99–3.01	–AdoH$^{\lambda}$ (**Mn**)
31.9/31.8	3.28–3.38	ProC$^{\beta}$–ProH$^{\delta,\delta'}$ (**Mn**)	35.1	1.19	AdoC$^{\alpha}$–AdoH$^{\gamma-\iota}$ (**Mj** and **Mn**)
35.1	1.44	AdoC$^{\alpha}$–AdoH$^{\beta}$ (**Mj** and **Mn**)	36.5–35.4	4.24–4.28	TyrC$^{\beta}$–TyrH$^{\alpha}$ (**Mj**)
36.5–35.4	4.38	AspC$^{\beta}$–AspH$^{\alpha}$ (**Mj**)	36.5–35.4	4.49	AspC$^{\beta}$–AspH$^{\alpha}$ (**Mn**)
36.5–35.4	7.02	TyrC$^{\beta}$–TyrArH^{meta} (**Mj** and **Mn**)	38.6/38.3	1.19	AdoC$^{\lambda}$–AdoH$^{\gamma-\iota}$ (**Mj** and **Mn**)
38.6/38.3	1.28	AdoC$^{\lambda}$–AdoH$^{\kappa}$ (**Mj**)	38.6/38.3	1.34	AdoC$^{\lambda}$–AdoH$^{\kappa}$ (**Mn**)

HMBC cross-peaks (continued)

38.6/38.3	7.23	AdoC$^{\lambda}$–AdoNH (**Mj**)	49.5	2.37	AspC$^{\alpha}$–AspH$^{\beta}$ (**Mj**)
49.5	2.54	AspC$^{\alpha}$–AspH$^{\beta}$ (**Mn**)	49.5	2.56	AspC$^{\alpha}$–AspH$^{\beta'}$ (**Mj**)
49.5	2.65	AspC$^{\alpha}$–AspH$^{\beta'}$ (**Mn**)	49.5	7.78	AspC$^{\alpha}$–AspNH (**Mj**)
54.3	2.64	TyrC$^{\alpha}$–TyrH$^{\beta}$ (**Mn**)	54.3	2.95	TyrC$^{\alpha}$–TyrH$^{\beta'}$ (**Mn**)
54.8	2.72	TyrC$^{\alpha}$–TyrH$^{\beta}$ (**Mj**)	54.8	2.89–2.92	TyrC$^{\alpha}$–TyrH$^{\beta'}$ (**Mj**)
54.8	7.81	TyrC$^{\alpha}$–TyrNH (**Mj**)	116.5/116.4	2.51	FlC^{9}–Fl8-CH_3 (**Mj** and **Mn**)
127.7/127.5	2.64	–TyrH$^{\beta}$ (**Mn**)	127.7/127.5	2.72	–TyrH$^{\beta}$ (**Mj**)
127.7/127.5	2.89–2.92	–TyrH$^{\beta'}$ (**Mj**)	127.7/127.5	2.95	–TyrH$^{\beta'}$ (**Mn**)
127.7/127.5	4.24–4.28	–TyrH$^{\alpha}$ (**Mj**)	127.7/127.5	6.44	–TyrArH^{ortho} (**Mn**)
127.7/127.5	6.62	–TyrArH^{ortho} (**Mj**)	130.0/129.9	2.64	–TyrH$^{\beta}$ (**Mn**)
130.0/129.9	2.72	–TyrH$^{\beta}$ (**Mj**)	130.0/129.9	2.89–2.92	–TyrH$^{\beta'}$ (**Mj**)
130.0/129.9	2.95	–TyrH$^{\beta'}$ (**Mn**)	130.9	2.41/2.40	FlC^{6}–Fl7-$C\mathit{H}_3$ (**Mj** and **Mn**)
131.7	4.01/4.00	FlC^{9a}–Fl10-CH_3	131.7	7.83	FlC^{9a}–Fl9-H
131.7	7.94	FlC^{9a}–Fl6-H	134.1/134.0	7.83	FlC^{5a}–Fl9-H
136.2–135.6	2.41/2.40	FlC^{7}–Fl7-CH_3	136.2–135.6	2.51	FlC^{7}–Fl8-CH_3
136.2–135.6	7.83	FlC^{7}–Fl9-H	147.3/147.0	2.41/2.40	FlC^{8}–Fl7-CH_3
147.3/147.0	2.51	FlC^{8}–Fl8-CH_3	147.3/147.0	7.94	FlC^{8}–Fl6-H
149.1/149.0	4.01/4.00	FlC^{10a}–Fl10-CH_3	154.2	3.93	FlC^{4}–Fl3-$C\mathit{H}_2$ (**Mn**)
154.5	4.72	FlC^{4}–Fl3-$C\mathit{H}_2$ (**Mj**)	154.5	4.78	FlC^{4}–Fl3-$C\mathit{H}_2$' (**Mj**)
155.8/155.6	6.44	TyrArC^{ipso}–TyrArH^{ortho} (**Mn**)	155.8/155.6	6.62	TyrArC^{ipso}–TyrArH^{ortho} (**Mj**)
155.8/155.6	7.02	TyrArC^{ipso}–TyrArH^{meta}	158.7	3.93	FlC^{2}–Fl3-$C\mathit{H}_2$ (**Mn**)
159.3	4.72	FlC^{2}–Fl3-$C\mathit{H}_2$ (**Mj**)	159.3	4.78	FlC^{2}–Fl3-$C\mathit{H}_2$' (**Mj**)
165.5	3.93	ProN*C*O–Fl3-$C\mathit{H}_2$(**Mn**)	166.2	4.72	ProN*C*O–Fl3-$C\mathit{H}_2$(**Mj**)
166.2	4.78	ProN*C*O–Fl3-$C\mathit{H}_2$'(**Mj**)	169.7	2.37	Asp*C*ONH–AspH$^{\beta}$ (**Mj**)
169.7	2.56	Asp*C*ONH–AspH$^{\beta'}$ (**Mj**)	169.7	2.89–2.92	Asp*C*ONH–AdoH$^{\lambda}$ (**Mj**)
169.7	4.38	Asp*C*ONH–AspH$^{\alpha}$ (**Mj**)	169.7	7.23	Asp*C*ONH–AdoNH (**Mj**)
169.8	2.54	Asp*C*ONH–AspH$^{\beta}$ (**Mn**)	169.8	2.65	Asp*C*ONH–AspH$^{\beta'}$ (**Mn**)
169.8	4.49	Asp*C*ONH–AspH$^{\alpha}$ (**Mn**)	169.8	7.57	Asp*C*ONH–AdoNH (**Mn**)
171.7–170.8	2.23	Pro*C*ONH–ProH$^{\beta'}$ (**Mn**)	171.7–170.8	2.37	Asp*C*$^{\beta}$O–AspH$^{\beta}$ (**Mj**)
171.7–170.8	2.56/2.54	Asp*C*$^{\beta}$O–AspH$^{\beta'}$ (**Mj**)<br>Asp*C*$^{\beta}$O–AspH$^{\beta}$ (**Mn**)	171.7–170.8	2.65	Asp*C*$^{\beta}$O–AspH$^{\beta'}$ (**Mn**)
171.7–170.8	2.72	Tyr*C*ONH–TyrH$^{\beta}$(**Mj**)	171.4–170.8	2.89–2.92	Tyr*C*ONH–TyrH$^{\beta'}$(**Mj**)
171.7–170.8	2.95	Tyr*C*ONH–TyrH$^{\beta'}$ (**Mn**)	171.4–170.8	4.24–4.28	Pro*C*ONH–ProH$^{\alpha}$(**Mj**)<br>Tyr*C*ONH–TyrH$^{\alpha}$(**Mj**)
171.7–170.8	4.38	Asp*C*$^{\beta}$O–AspH$^{\alpha}$ (**Mj**)<br>Tyr*C*ONH–AspH$^{\alpha}$(**Mj**)	171.7–170.8	4.49	Asp*C*$^{\beta}$O–AspH$^{\alpha}$ (**Mn**)<br>Tyr*C*ONH–AspH$^{\alpha}$ (**Mn**)
171.4–170.8	7.78	Tyr*C*ONH–AspNH (**Mj**)	171.4–170.8	7.81	Pro*C*ONH–TyrNH (**Mj**)
171.4–170.8	8.21	Tyr*C*ONH–AspNH (**Mn**)	171.4–170.8	8.40	Pro*C*ONH–TyrNH (**Mn**)
174.3	1.44	*C*ONH_2–AdoH$^{\beta}$ (**Mj** and **Mn**)	174.3	1.99/2.00	*C*ONH_2–AdoH$^{\alpha}$ (**Mj** and **Mn**)

ROESY cross-peaks

F1 (ppm)	F2 (ppm)	Assignment	F1 (ppm)	F2 (ppm)	Assignment
1.19	1.44	AdoH$^{\gamma-\iota}$–AdoH$^{\beta}$	1.19	1.99/2.00	AdoH$^{\gamma-\iota}$–AdoH$^{\alpha}$
1.44	1.19	(**Mj** and **Mn**)	1.99/2.00	1.19	(**Mj** and **Mn**)
1.19	2.89–2.92	AdoH$^{\gamma-\iota}$–AdoH$^{\lambda}$ (**Mj**)	1.19	2.99–3.01	AdoH$^{\gamma-\iota}$–AdoH$^{\lambda}$ (**Mn**)
2.89–2.92	1.19		2.99–3.01	1.19	
1.28	2.89–2.92	AdoH$^{\kappa}$–AdoH$^{\lambda}$ (**Mj**)	1.28	7.23	AdoH$^{\kappa}$–AdoNH (**Mj**)
2.89–2.92	1.28		7.23	1.28	
1.34	2.99–3.01	AdoH$^{\kappa}$–AdoH$^{\lambda}$ (**Mn**)	1.34	7.57	AdoH$^{\kappa}$–AdoNH (**Mn**)
2.99–3.01	1.34		7.57	1.34	
1.44	1.99/2.00	AdoH$^{\beta}$–AdoH$^{\alpha}$	1.44	6.65	AdoH$^{\beta}$–CONH_2 (**Mj**)
1.99/2.00	1.44	(**Mj** and **Mn**)	6.65	1.44	
1.44	7.19	AdoH$^{\beta}$–CONH_2 (**Mn**)	1.68–1.79	1.87–1.90	ProH$^{\beta}$/ProH$^{\gamma}$–ProH$^{\gamma'}$(**Mj**)
7.19	1.44		1.87–1.90	1.68–1.79	ProH$^{\gamma}$/ProH$^{\gamma'}$–ProH$^{\beta}$(**Mn**)
1.68–1.79	1.96	ProH$^{\beta}$/ProH$^{\gamma}$–ProH$^{\beta'}$(**Mj**)	1.68–1.79	2.23	ProH$^{\gamma}$/ProH$^{\gamma'}$–ProH$^{\beta'}$ (**Mn**)
1.96	1.68–1.79		2.23	1.68–1.79	
1.68–1.79	3.28–3.38	ProH$^{\gamma}$/ProH$^{\gamma'}$–ProH$^{\delta,\delta'}$	1.68–1.79	3.61–3.71	ProH$^{\beta}$/ProH$^{\gamma}$–ProH$^{\delta,\delta'}$(**Mj**)
3.28–3.38	1.68–1.79	(**Mn**)	3.61–3.71	1.68–1.79	
1.68–1.79	4.24–4.28	ProH$^{\beta}$/ProH$^{\gamma}$–ProH$^{\alpha}$ (**Mj**)	1.68–1.79	7.02	–TyrArH^{meta}
4.24–4.28	1.68–1.79		7.02	1.68–1.79	(**Mj** and **Mn**)
1.68–1.79	7.81	ProH$^{\beta}$/ProH$^{\gamma}$–TyrNH	1.87–1.90	2.23	ProH$^{\beta}$–ProH$^{\beta'}$ (**Mn**)
7.81	1.68–1.79	(**Mj**)	2.23	1.87–1.90	
1.87–1.90	3.61–3.71	ProH$^{\gamma'}$–ProH$^{\delta,\delta'}$ (**Mj**)	1.87–1.90	4.24–4.28	ProH$^{\gamma'}$–ProH$^{\alpha}$ (**Mj**)
3.61–3.71	1.87–1.90		4.24–4.28	1.87–1.90	
1.87–1.90	8.40	ProH$^{\beta}$–TyrNH (**Mn**)	1.96	7.81	ProH$^{\beta'}$–TyrNH (**Mj**)
8.40	1.87–1.90		7.81	1.96	
1.99/2.00	4.24–4.28	AdoH$^{\alpha}$–ProH$^{\alpha}$/TyrH$^{\alpha}$	1.99/2.00	6.65	AdoH$^{\alpha}$–CONH_2 (**Mj**)
4.24–4.28	1.99/2.00	(**Mj**)	6.65	1.99/2.00	
1.99/2.00	7.19	AdoH$^{\alpha}$–CONH_2 (**Mn**)	2.23	3.28–3.38	ProH$^{\beta'}$–ProH$^{\delta/\delta'}$ (**Mn**)
7.19	1.99/2.00		3.28–3.38	2.23	
2.23	3.93	ProH$^{\beta'}$–Fl3-CH$_2$ (**Mn**)	2.23	4.43	ProH$^{\beta'}$–ProH$^{\alpha}$ (**Mn**)
3.93	2.23		4.43	2.23	
2.23	8.40	ProH$^{\beta'}$–TyrNH (**Mn**)	2.37	2.56	AspH$^{\beta}$–AspH$^{\beta'}$ (**Mj**)
8.40	2.23		2.56	2.37	
2.37	4.38	AspH$^{\beta}$–AspH$^{\alpha}$ (**Mj**)	2.37	7.78	AspH$^{\beta}$–AspNH (**Mj**)
4.38	2.37		7.78	2.37	
2.40/2.41	7.94	Fl7-CH$_3$–Fl6-H	2.51	4.00/4.01	Fl8-CH$_3$–Fl10-CH$_3$
7.94	2.40/2.41	(**Mj** and **Mn**)	4.00/4.01	2.51	(**Mj** and **Mn**)
2.51	7.83	Fl8-CH$_3$–Fl9-H	2.54	4.49	AspH$^{\beta}$–AspH$^{\alpha}$ (**Mn**)
7.83	2.51	(**Mj** and **Mn**)	4.49	2.54	
2.54	8.21	AspH$^{\beta}$–AspNH (**Mn**)	2.56	4.38	AspH$^{\beta'}$–AspH$^{\alpha}$ (**Mj**)
8.21	2.54		4.38	2.56	
2.64/2.65	2.95	TyrH$^{\beta}$–TyrH$^{\beta'}$ (**Mn**)	2.64/2.65	4.49	AspH$^{\beta'}$–AspH$^{\alpha}$ (**Mn**)
2.95	2.64/2.65		4.49	2.64/2.65	
2.64/2.65	7.02	TyrH$^{\beta}$–TyrArH^{meta} (**Mn**)	2.64/2.65	8.40	TyrH$^{\beta}$–TyrNH (**Mn**)
7.02	2.64/2.65		8.40	2.64/2.65	
2.72	2.89–2.92	TyrH$^{\beta}$–TyrH$^{\beta'}$ (**Mj**)	2.72	7.02	TyrH$^{\beta}$–TyrArH^{meta} (**Mj**)
2.89–2.92	2.72		7.02	2.72	
2.72	7.81	TyrH$^{\beta}$–Tyr-NH (**Mj**)	2.89–2.92	4.24–4.28	TyrH$^{\beta'}$–TyrH$^{\alpha}$ (**Mj**)
7.81	2.72		4.24–4.28	2.89–2.92	
2.89–2.92	7.02	TyrH$^{\beta'}$–TyrArH^{meta} (**Mj**)	2.89–2.92	7.23	AdoH$^{\lambda}$–AdoNH (**Mj**)
7.02	2.89–2.92		7.23	2.89–2.92	
2.89–2.92	7.81	TyrH$^{\beta'}$–Tyr-NH (**Mj**)	2.95	4.53	TyrH$^{\beta'}$– TyrH$^{\alpha}$ (**Mn**)
7.81	2.89–2.92		4.53	2.95	

ROESY cross-peaks (continued)

F1 (ppm)	F2 (ppm)	Assignment	F1 (ppm)	F2 (ppm)	Assignment
2.95<br>7.02	7.02<br>2.95	TyrH$^{\beta'}$–TyrArH^{meta} (**Mn**)	2.95<br>8.40	8.40<br>2.95	TyrH$^{\beta'}$–TyrNH (**Mn**)
2.99–3.01<br>7.57	7.57<br>2.99–3.01	AdoH$^{\lambda}$–AdoNH (**Mn**)	3.61–3.71<br>4.72	4.72<br>3.61–3.71	ProH$^{\delta,\delta'}$–Fl3-CH_2 (**Mj**)
3.61–3.71<br>4.78	4.78<br>3.61–3.71	ProH$^{\delta,\delta'}$–Fl3-CH_2' (**Mj**)	3.93<br>4.43	4.43<br>3.93	Fl3-CH_2–ProH$^{\alpha}$ (**Mn**)
3.93<br>6.44	6.44<br>3.93	Fl3-CH_2–TyrArH^{ortho} (**Mn**)	3.93<br>7.02	7.02<br>3.93	Fl3-CH_2– TyrArH^{meta} (**Mn**)
3.93<br>8.40	8.40<br>3.93	Fl3-CH_2–TyrNH (**Mn**)	4.00/4.01<br>7.83	7.83<br>4.00/4.01	Fl10-CH_3–Fl9-H (**Mj** and **Mn**)
4.24–4.28<br>7.02	7.02<br>4.24–4.28	TyrH$^{\alpha}$ –TyrArH^{meta} (**Mj**)	4.24–4.28<br>7.78	7.78<br>4.24–4.28	TyrH$^{\alpha}$ –AspNH (**Mj**)
4.24–4.28<br>7.81	7.81<br>4.24–4.28	ProH$^{\alpha}$–TyrNH (**Mj**)	4.38<br>7.23	7.23<br>4.38	AspH$^{\alpha}$–AdoNH (**Mj**)
4.38<br>7.78	7.78<br>4.38	AspH$^{\alpha}$–AspNH (**Mj**)	4.43<br>8.40	8.40<br>4.43	ProH$^{\alpha}$–TyrNH (**Mn**)
4.49<br>7.57	7.57<br>4.49	AspH$^{\alpha}$–AdoNH (**Mn**)	4.53<br>6.44	6.44<br>4.53	TyrH$^{\alpha}$–TyrArH^{ortho} (**Mn**)
4.53<br>7.02	7.02<br>4.53	TyrH$^{\alpha}$–TyrArH^{meta} (**Mn**)	4.53<br>8.21	8.21<br>4.53	TyrH$^{\alpha}$–AspNH (**Mn**)
4.53<br>8.40	8.40<br>4.53	TyrH$^{\alpha}$–TyrNH (**Mn**)	6.44<br>7.02	7.02<br>6.44	TyrArH^{ortho}–TyrArH^{meta} (**Mn**)
6.62<br>7.02	7.02<br>6.62	TyrArH^{ortho}–TyrArH^{meta} (**Mj**)	7.02<br>7.81	7.81<br>7.02	TyrArH^{meta}–TyrNH (**Mj**)
7.02<br>8.40	8.40<br>7.02	TyrArH^{meta}–TyrNH (**Mn**)	7.23<br>7.78	7.78<br>7.23	AdoNH–AspNH (**Mj**)
7.57<br>8.21	8.21<br>7.57	AdoNH–AspNH (**Mn**)			

ROESY map

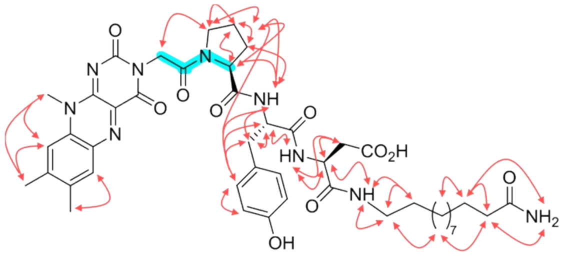

Mj (*trans* of 3-FlC2-Pro amide bond)

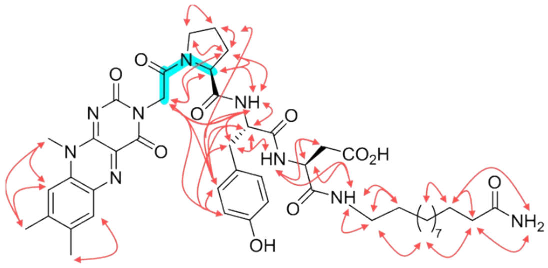

Mn (*cis* of 3-FlC2-Pro amide bond)

6. Aerobic oxidation of thioanisole with flavin catalyst

6a. Typical procedure

To a stirred mixture of thioanisole (12.4 mg, 0.10 mmol) and flavin catalyst (10 µmol, 10 mol%) in a TFE—DCE mixed solvent (1:1, 0.5 mL) was added $NH_2NH_2 \cdot H_2O$ (20 mg, 0.40 mmol), and the resulting mixture was continued to stir at 25 °C for 24–36 h under an atmosphere of oxygen. The yield of methyl phenyl sulfoxide was determined by means of GC analysis with the corrected area normalization method.

6b. Comparison under different conditions

Reactions were carried out under several different conditions, whose results were summarized below.

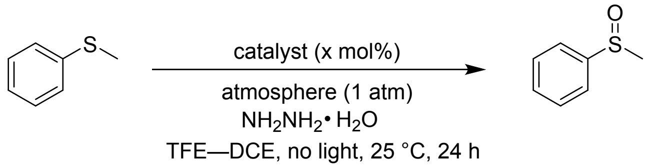

Entry	Catalyst (loading [mol%])	atmosphere	Reductant (amount [equiv])	TFE—DCE	Yield (%)[a]
1	**Fl-Pep5-b** (10)	O_2	$NH_2NH_2 \cdot H_2O$ (4)	100:0	26
2	**Fl-Pep5-b** (10)	O_2	$NH_2NH_2 \cdot H_2O$ (4)	75:25	40
3	**Fl-Pep5-b** (10)	O_2	$NH_2NH_2 \cdot H_2O$ (4)	50:50	62
4	**Fl-Pep5-b** (10)	O_2	$NH_2NH_2 \cdot H_2O$ (4)	25:75	4
5	**Fl-Pep5-b** (10)	air	$NH_2NH_2 \cdot H_2O$ (4)	50:50	15
6	**Fl-Pep5-b** (10)	N_2	$NH_2NH_2 \cdot H_2O$ (4)	50:50	0
7	**Fl-Pep5-b** (5)	O_2	$NH_2NH_2 \cdot H_2O$ (4)	50:50	50
8	**Fl-Pep5-b** (10)	O_2	$NH_2NH_2 \cdot H_2O$ (0)	50:50	0
9	**Fl-Pep1-a** (10)	O_2	$NH_2NH_2 \cdot H_2O$ (2)	50:50	6
10	**Fl-Pep1-a** (10)	O_2	$NH_2NH_2 \cdot H_2O$ (4)	50:50	36
11	**Fl-Pep1-a** (10)	O_2	$NH_2NH_2 \cdot H_2O$ (6)	50:50	57
12	**Fl-Pep1-a** (10)	O_2	$NH_2NH_2 \cdot H_2O$ (10)	50:50	92
13	**3-FlC2-Pro-Tyr-Asp-Ado-NH$_2$** (10)	O_2	$NH_2NH_2 \cdot H_2O$ (4)	50:50	7
14	**3-FlC2-Pro-Tyr-Asp-Ado-NH$_2$** (10)	O_2	$NH_2NH_2 \cdot H_2O$ (8)	50:50	40
15	**Fl-Pep5-b** (10)	O_2	50% NH_2OH aq. (5)	50:50	3 (35)[b]

[a] Determined by GC analysis. [b] Yield after heating at 60 °C for another 24 h.

6c. Product isolation

To a stirred mixture of thioanisole (124 mg, 1.0 mmol) and **Fl-Pep-b** (5 μmol, 5 mol%) in a TFE–DCE mixed solvent (1:1, 5 mL) was added $NH_2NH_2 \cdot H_2O$ (200 mg, 4.0 mmol), and the resulting mixture was further stirred at 25 °C under an atmosphere of oxygen. After 14 h $NH_2NH_2 \cdot H_2O$ (200 mg, 4.0 mmol) was added, and the reaction was continued for another 34 h to give methyl phenyl sulfoxide in 97% GC yield. The catalyst was filtered out and washed with CH_2Cl_2 (5 × 1 mL), and the combined filtrate was washed with saturated aqueous Na_2SO_3 (5 mL). The aqueous layer was extracted with CH_2Cl_2 (2 × 10 mL), and the combined organic layers were dried over Na_2SO_4, which was filtered and concentrated under reduced pressure. The resulting crude product was purified by flash column chromatography on silica gel using a gradient of hexane:EtOAc from 8:1 to 2:3 to afford 119 mg of methyl phenyl sulfoxide as colorless oil (85%). Analytical data were in agreement with the published data.[7]

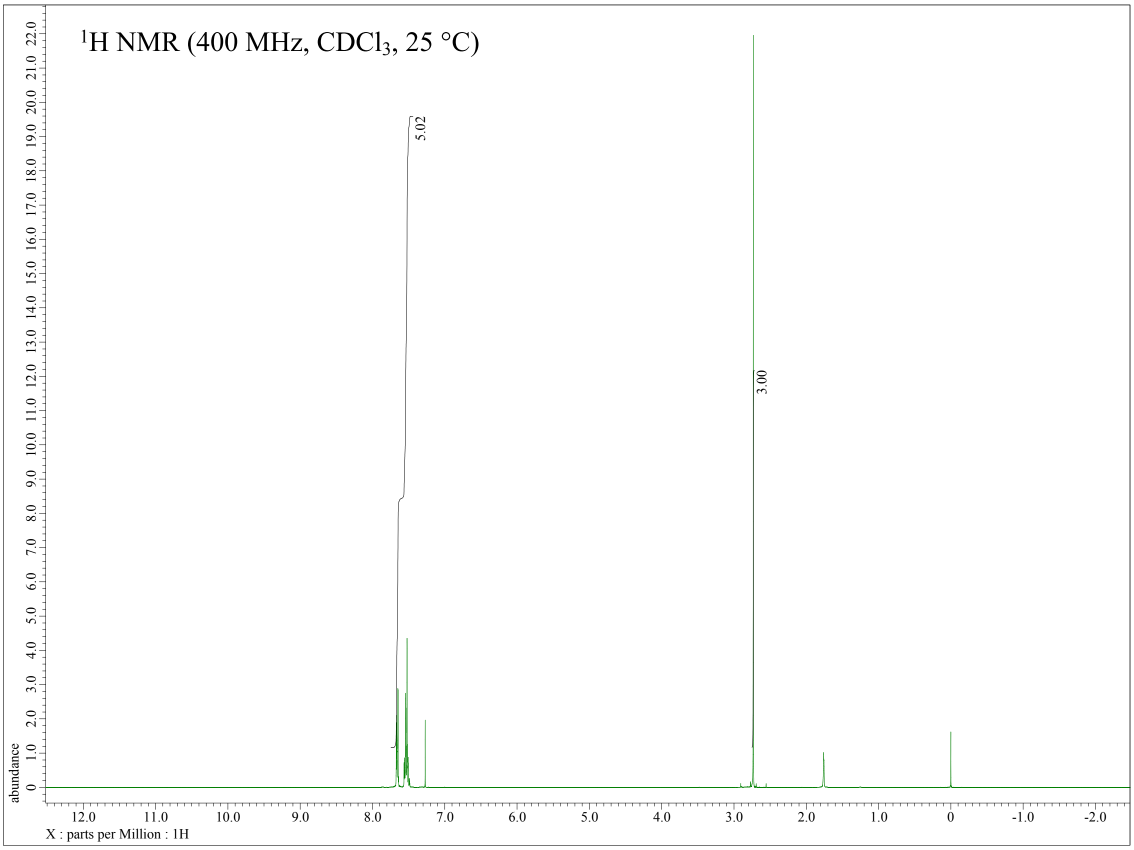

6d. Competitive reaction of *p*-substituted methyl phenyl sulfides for Fl-Pep1-a-catalyzed aerobic oxygenation

To a stirred mixture of thioanisole (10 mg, 0.08 mmol), *p*-substituted methyl phenyl sulfide (0.08 mmol), and **Fl-Pep1-a** (34 mg, 16 μmol, 10 mol%) in a TFE—DCE mixed solvent (1:1, 0.8 mL) was added $NH_2NH_2 \cdot H_2O$ (32 mg, 0.64 mmol), and the resulting mixture was continued to stir at 25 °C for 4 h under an atmosphere of oxygen. Saturated aqueous Na_2SO_3 was added to quench the reaction, and the reaction mixture was extracted with diethyl ether. The combined extracts were dried over $MgSO_4$ and evaporated under reduced pressure. The product ratios were determined on the basis of the integration of the methyl protons (X-$C_6H_4S(O)CH_3$). The singlets for methyl protons of X-$C_6H_4SOCH_3$ are observed at δ = 2.71 (*p*-MeO), 2.71 (*p*-Me), 2.73 (*p*-H), 2.75 (*p*-CN), and 2.78 ppm (*p*-NO_2).

7. Aerobic Baeyer-Villiger oxidation of 3-phenylcyclobutan-1-one with flavin catalyst

7a. Typical procedure

A mixture of 3-phenylcyclobutanone (14.6 mg, 0.10 mmol), flavin catalyst (5.0 μmol, 5 mol%), zinc (22.8 mg, 0.35 mmol), H_2O (36 μl, 2.0 mmol), and dodecane (1 μmol, internal standard) in an acetonitrile—toluene—ethyl acetate mixed solvent (8:4:1, 1.0 mL) was stirred at a defined temperature (25–60 ^{o}C) for 6–40 h under an atmosphere of oxygen. The yield of β-phenyl-γ-butyrolactone was determined by ^{1}H NMR spectroscopy of the crude mixture.

7b. Comparison under different conditions

Reactions were carried out under several different conditions, whose results were summarized below.

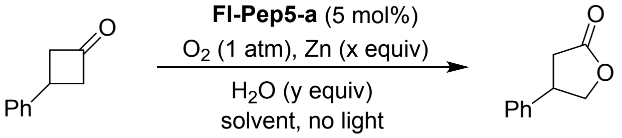

Entry	Solvent	Zinc (equiv)	H_2O (equiv)	Temp. (°C)	Time (h)	Yield (%)[a]
1	EtOH/Toluene 1:1	5	0	60	6	66
2	Toluene	5	0	60	24	<1
3	EtOH	5	0	60	24	2
4[b]	EtOH/Toluene 1:1	5	0	60	24	<1
5	EtOH/Toluene 1:1	0	0	60	24	<1
6[c]	EtOH/Toluene 1:1	5	0	60	24	<1
7	CH_3CN/Toluene 1:1	5	10	60	6	35
8	CH_3CN/Toluene 1:1	5	50	60	6	11
9	CH_3CN/Toluene 1:1	5	100	60	6	3
10	CH_3CN/Toluene 2:1	5	5	60	6	5
11	CH_3CN/Toluene 2:1	5	30	60	6	56
12	CH_3CN/Toluene 2:1	5	50	60	12	61
13	CH_3CN/Toluene 2:1	5	10	35	40	70
14	CH_3CN/Toluene 2:1	5	10	25	40	42
15	CH_3CN/Toluene 2:1	5	30	35	24	65
16	CH_3CN/Toluene 2:1	3.5	20	35	24	60
17[d]	CH_3CN/Toluene/EtOAc 8:4:1	3.5	20	35	7	72
18[d]	CH_3CN/Toluene/EtOAc 8:4:1	3.5	0	35	7	18
19[d]	CH_3CN/Toluene/EtOAc 8:4:1	2	20	35	7	19
20[d]	CH_3CN/Toluene/EtOAc 8:4:1	3.5	20	35	4	58
21[d]	CH_3CN/Toluene/EtOAc 4:2:1	3.5	20	35	4	47
22[d]	CH_3CN/Toluene/EtOAc 2:1:1	3.5	20	35	4	31
23[d]	CH_3CN/Toluene/EtOAc 8:2:1	3.5	20	35	4	57

[a] Determined by ^1H NMR analysis using hexadecane or dodecane as an internal standard. [b] In the absence of the catalyst. [c] Under N_2. [d] **Fl-Pep5-b** was used instead of **Fl-Pep5-a**.

7c. Product isolation

A mixture of 3-phenylcyclobutanone (117 mg, 0.80 mmol), **Fl-Pep5-b** (0.040 mmol, 5 mol%), H_2O (290 μL, 16 mmol) and zinc (183 mg, 2.80 mmol) in an acetonitrile—toluene—ethyl acetate mixed solvent (8:4:1, 8.0 mL) was stirred at 35 °C under an atmosphere of oxygen for 15 h. The catalyst was filtered out and washed with CH_2Cl_2 (5 × 1 mL), and the combined filtrate was washed with saturated aqueous Na_2SO_3 (5 mL). The aqueous layer was extracted with CH_2Cl_2 (4 × 7 mL), and the combined organic layers were dried over Na_2SO_4, which was filtered and concentrated under reduced pressure. The resulting crude product was purified by flash column chromatography on silica gel using a mixture of hexane and ethyl acetate 7:3 as eluent to afford 83 mg of β-phenyl-γ-butyrolactone as colorless oil (66%). Analytical data were in agreement with the published data.[8] ^{1}H NMR (400 MHz, $CDCl_3$, δ) : 2.68 (dd, J = 9.1, 17.4 Hz, 1 H, -C(O)C*H*H-), 2.93 (dd, J = 8.7, 17.4 Hz, 1 H, -C(O)CH*H*-), 3.76-3.83 (m, 1 H, ArC*H*), 4.27 (dd, J = 8.0, 9.2 Hz, 1 H, -OCH*H*-), 4.67 (dd, J = 7.9, 9.1 Hz, 1 H, -OCH*H*-), 7.23-7.25 (m, 2 H, Ar*H*), 7.29-7.32 (m, 1 H, Ar*H*), 7.36-7.39 (m, 2 H, Ar*H*); ^{13}C NMR ($CDCl_3$, 100 MHz, δ) : 35.7, 41.1, 74.1, 126.7, 127.7, 129.2, 139.5, 176.4; Anal. calcd. for $C_{10}H_{10}O_2$: C 74.06, H 6.22; found: C 73.97, H 6.30.

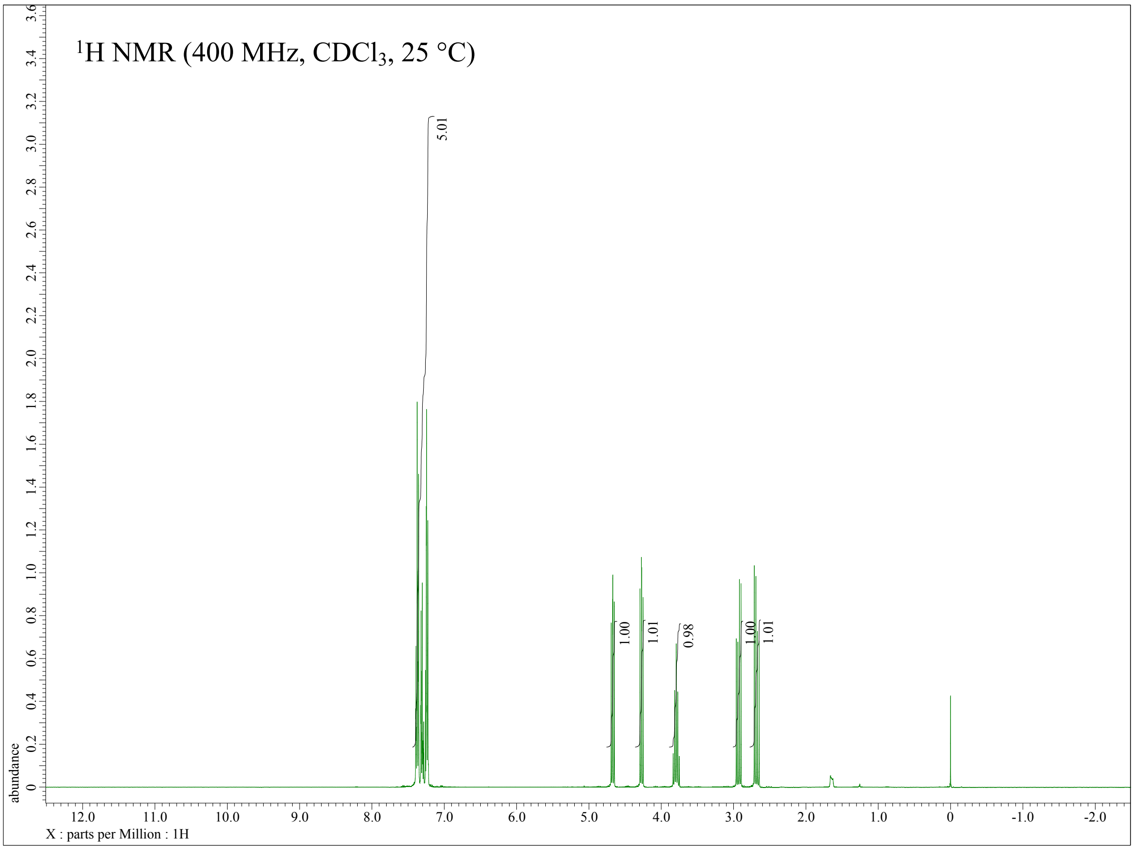

7d. Competitive reaction

Competitive experiments (equations 1–4 in the main text) were carried out and analyzed according to the following procedures.

3-phenylcyclobutanone vs cyclooctene under Fl-Pep conditions (equation 1)

A mixture of 3-phenylcyclobutanone (14.6 mg, 0.10 mmol), cyclooctene (11.0 mg, 0.10 mmol), **Fl-Pep5-b** (5.0 μmol, 5 mol%), dodecane (0.010 mmol, internal standard), H_2O (36 μL, 2.0 mmol), and zinc (22.9 mg, 0.35 mmol) in an acetonitrile—toluene—ethyl acetate mixed solvent (8:4:1, 1.0 mL) was stirred at 35 $^{\circ}C$ for 7 h under an atmosphere of oxygen. The yields of products were determined by ^{1}H NMR spectroscopy of the crude mixture (see below).

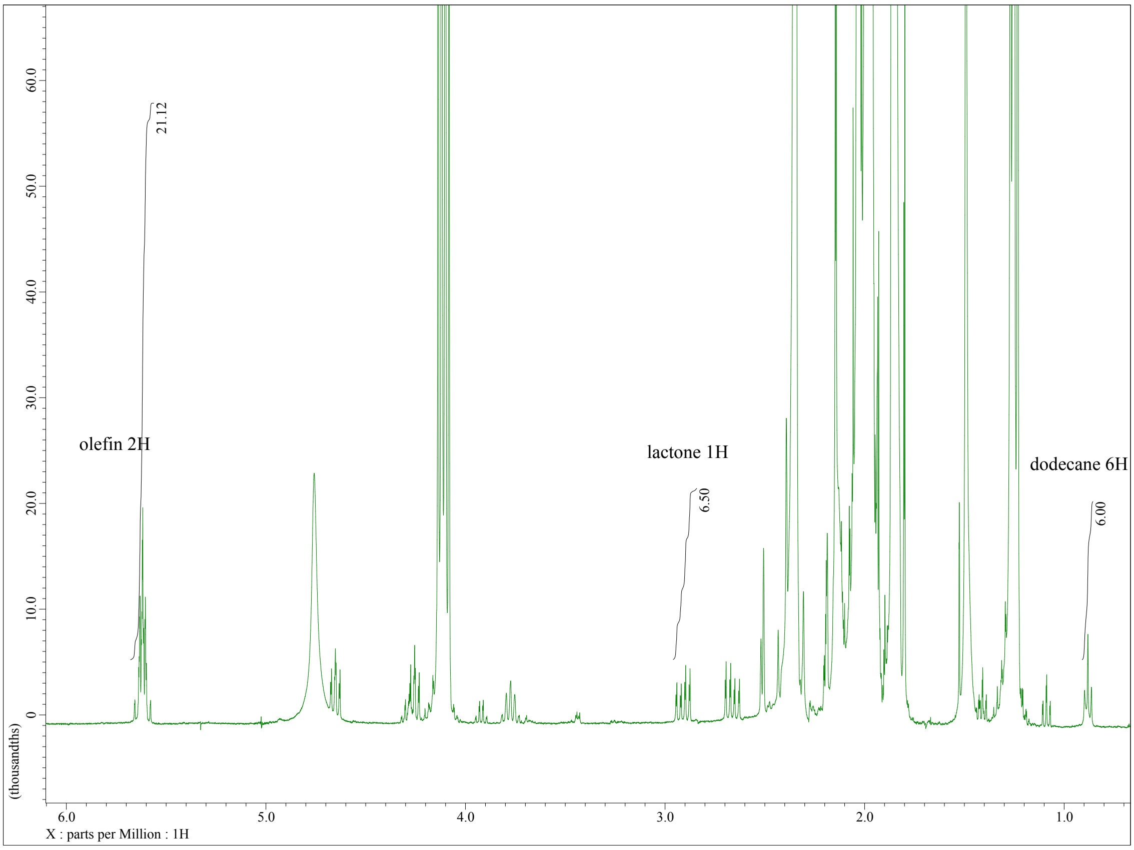

	chemical shift (ppm)	peak integral	yield (%)
dodecane (6*H*)	0.88	6.00	-
lactone (1*H*)	2.93	6.50	67
epoxide (2*H*)	2.90	0	0

lactone = β-phenyl-γ-butyrolactone, epoxide = cyclooctene oxide

3-phenylcyclobutanone vs cyclooctene under mCPBA conditions (equation 2)

A mixture of 3-phenylcyclobutanone (14.6 mg, 0.10 mmol), cyclooctene (11.0 mg, 0.10 mmol), *m*CPBA (20.9 mg, 0.12 mmol), $NaHCO_3$ (8.4 mg, 0.10 mmol), and dodecane (0.010 mmol, internal standard) in CH_2Cl_2 (0.5 mL) was stirred at room temperature for 24 h. The yields of products were determined by ^1H NMR spectroscopy of the crude mixture (see below).

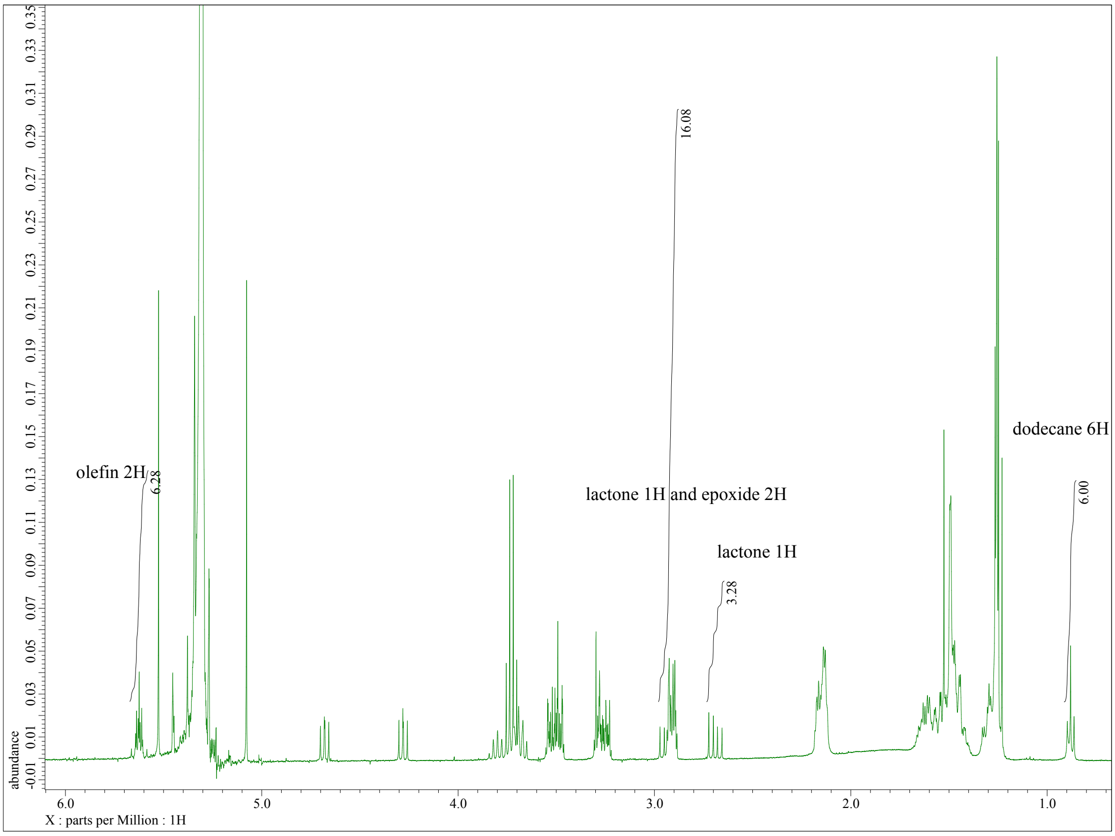

	chemical shift (ppm)	peak integral	yield (%)
dodecane (6*H*)	0.88	6.00	-
lactone (1*H*)	2.65–2.71	3.28	33
lactone (1*H*) and epoxide (2*H*)	2.88–2.97	16.08	-
epoxide (2*H*)	2.88–2.94	16.08–3.28 = 12.8	64

lactone = β-phenyl-γ-butyrolactone, epoxide = cyclooctene oxide

3-phenylcyclobutanone vs methyl phenyl sulfide under Fl-Pep conditions (equation 3)

A mixture of 3-phenylcyclobutanone (14.6 mg, 0.10 mmol), methyl phenyl sulfide (12.4 mg, 0.10 mmol), **Fl-Pep5-b** (5.0 μmol, 5 mol%), dodecane (0.010 mmol, internal standard), H_2O (36 μL, 2.0 mmol), and zinc (22.9 mg, 0.35 mmol) in an acetonitrile—toluene—ethyl acetate mixed solvent (8:4:1, 1.0 mL) was stirred at 35 ^{o}C for 7 h under an atmosphere of oxygen. The yields of products were determined by ^{1}H NMR spectroscopy of the crude mixture (see below).

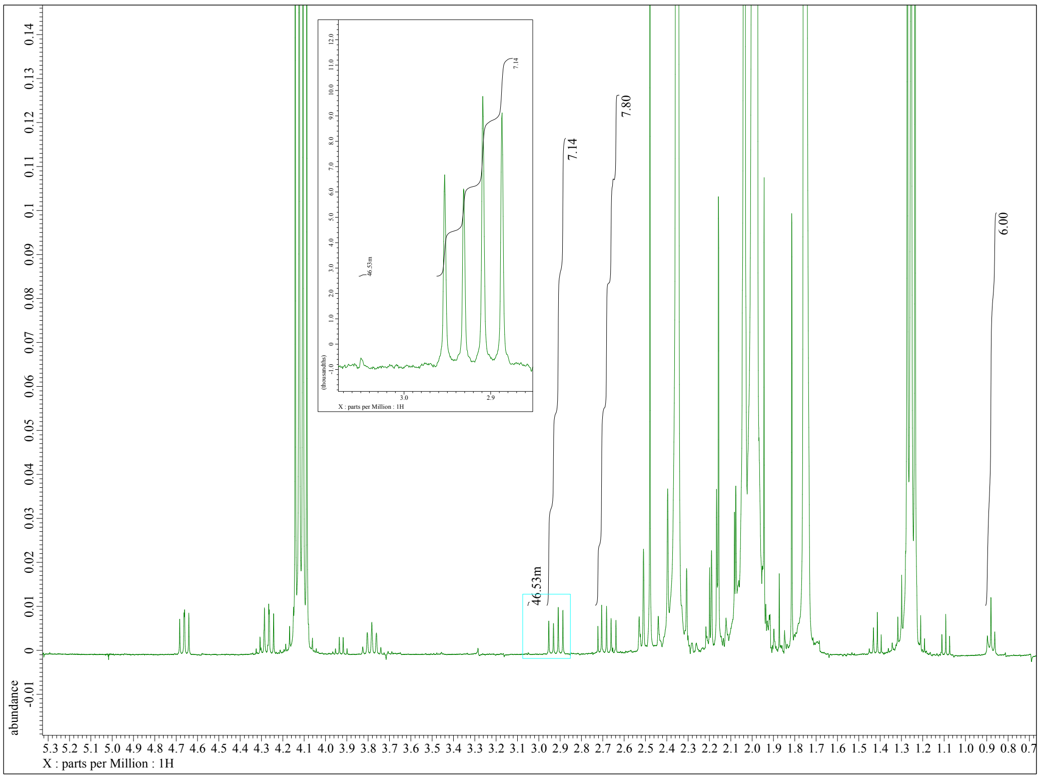

	chemical shift (ppm)	peak integral	yield (%)
dodecane (6*H*)	0.88	6.00	-
lactone (1*H*) and sulfoxide (3*H*)	2.65–2.73	7.80	-
lactone (1*H*)	2.93	7.14	65
sulfoxide (3*H*)	2.73	7.80–7.14 = 0.66	2
sulfone (3*H*)	3.05	0.046	<1

lactone = β-phenyl-γ-butyrolactone, sulfoxide = methyl phenyl sulfoxide, sulfone = methyl phenyl sulfone

3-phenylcyclobutanone vs methyl phenyl sulfide under mCPBA conditions (equation 4)

A mixture of 3-phenylcyclobutanone (14.6 mg, 0.10 mmol), methyl phenyl sulfide (12.4 mg, 0.10 mmol), *m*CPBA (20.9 mg, 0.12 mmol), $NaHCO_3$ (8.4 mg, 0.10 mmol), and dodecane (0.010 mmol, internal standard) in CH_2Cl_2 (0.5 mL) was stirred at room temperature for 24 h. The yields of products were determined by ^{1}H NMR spectroscopy of the crude mixture (see below).

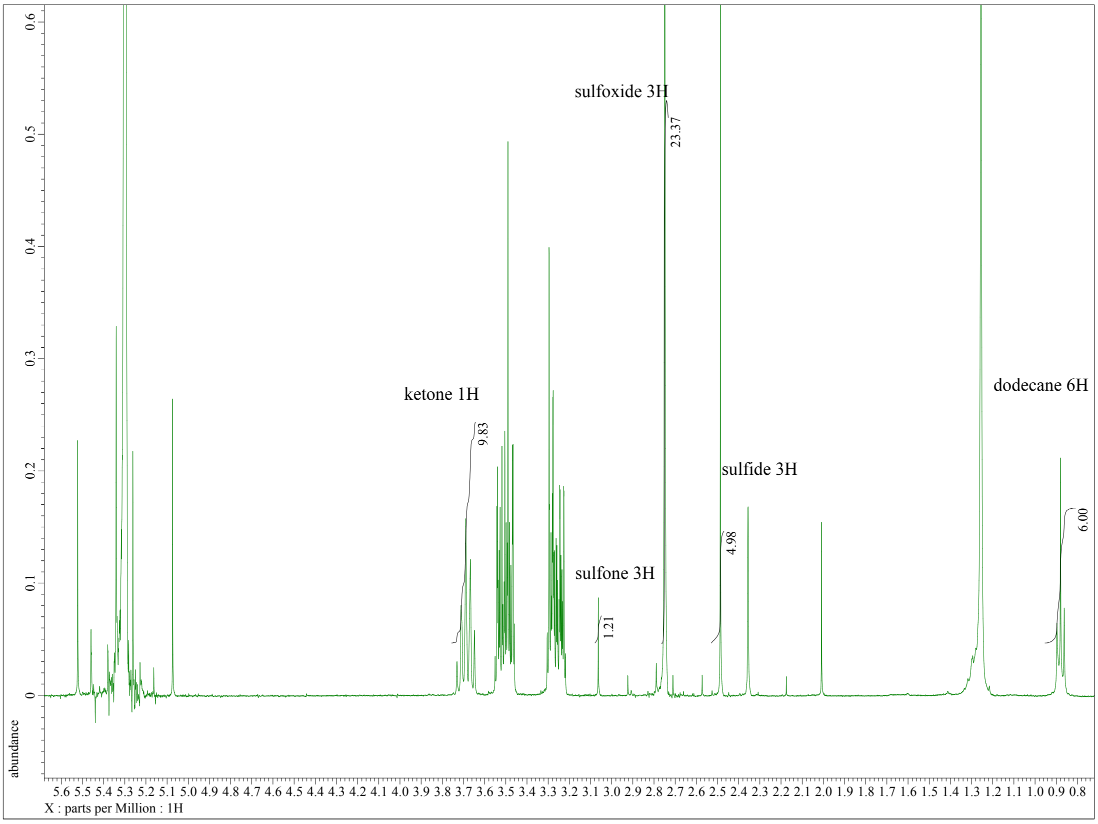

	chemical shift (ppm)	peak integral	yield (%)
dodecane (6*H*)	0.88	6.00	-
lactone (1*H*)	2.93	0	0
sulfoxide (3*H*)	2.75	23.37	78
sulfone (3*H*)	3.05	1.21	4

lactone = β-phenyl-γ-butyrolactone, sulfoxide = methyl phenyl sulfoxide, sulfone = methyl phenyl sulfone

8. NMR spectra for 3-FlC2-Pro-Tyr-Asp-Ado-NH_2

^{1}H NMR

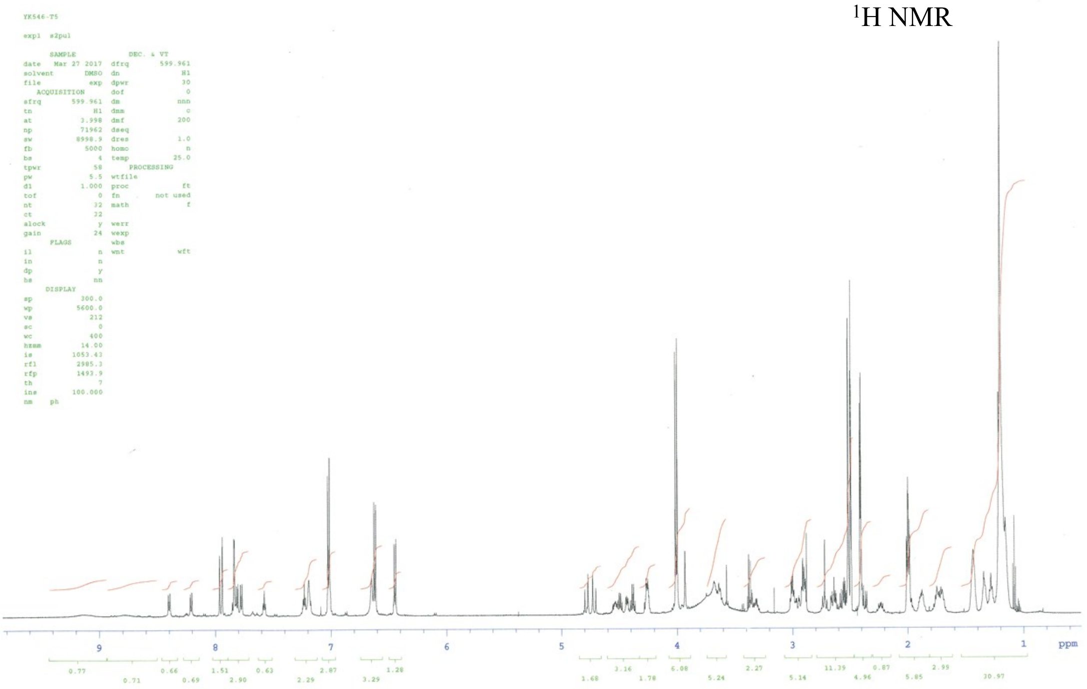

^{13}C NMR

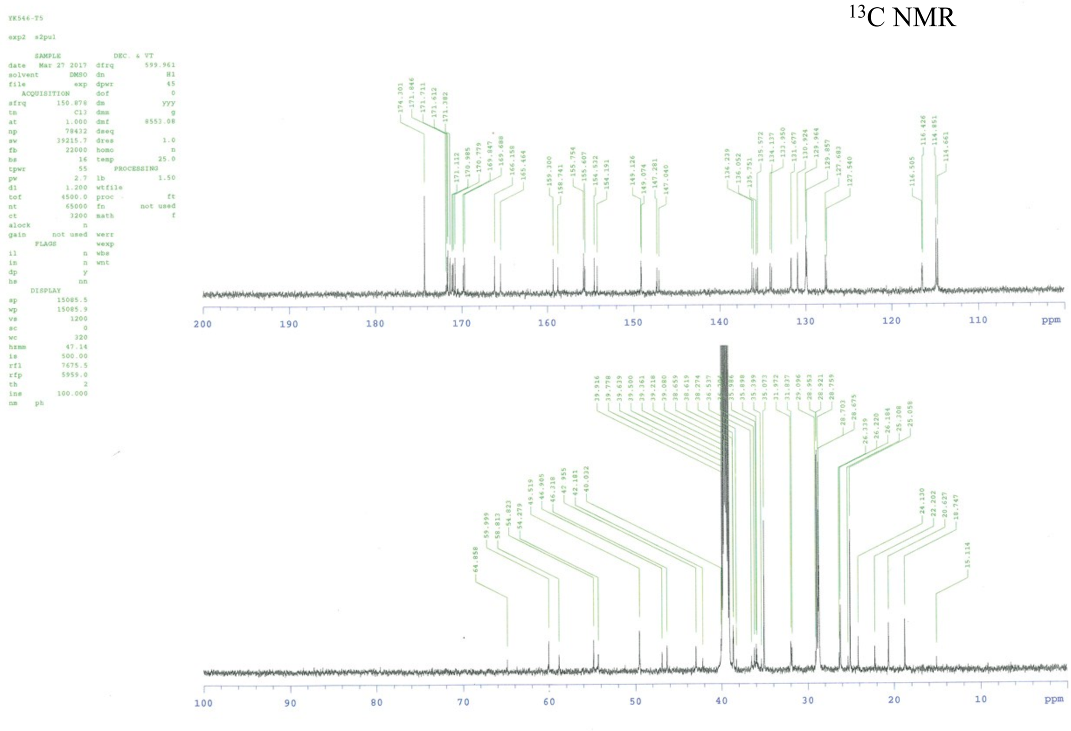

COSY

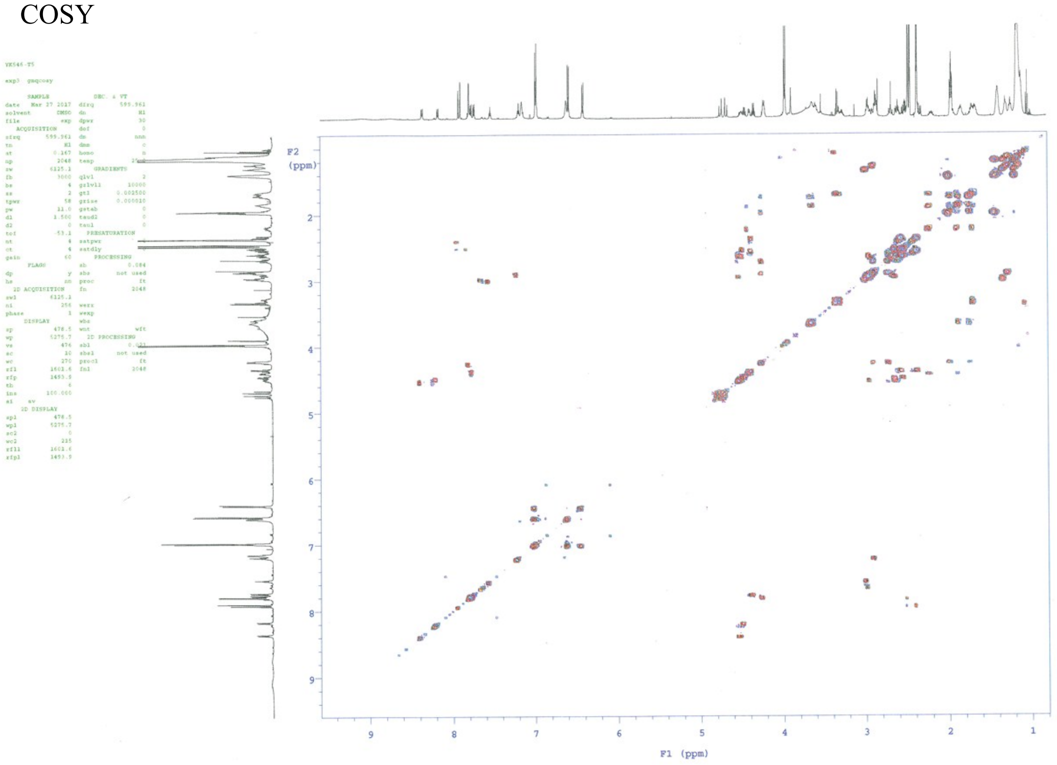

HMQC

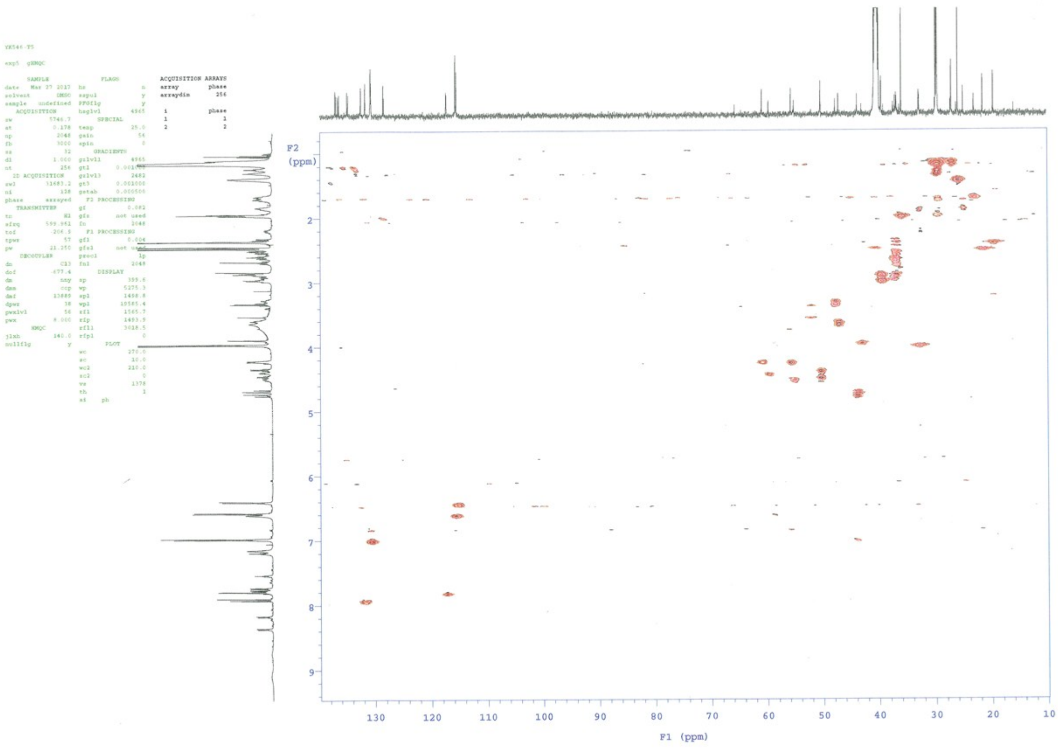

HMBC

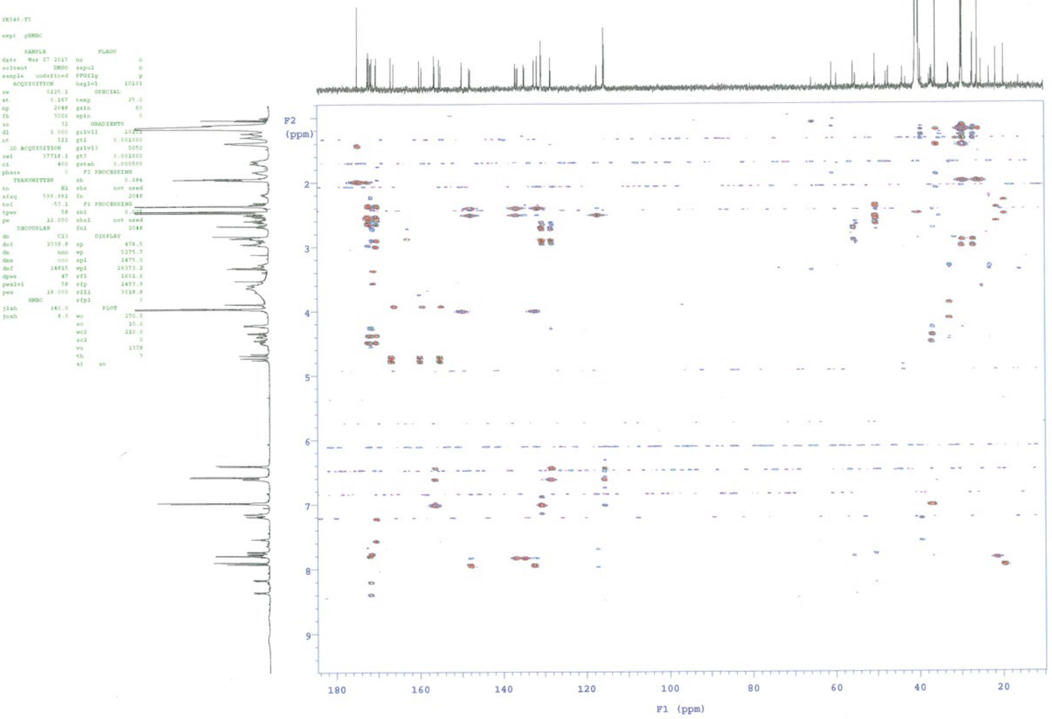

ROESY

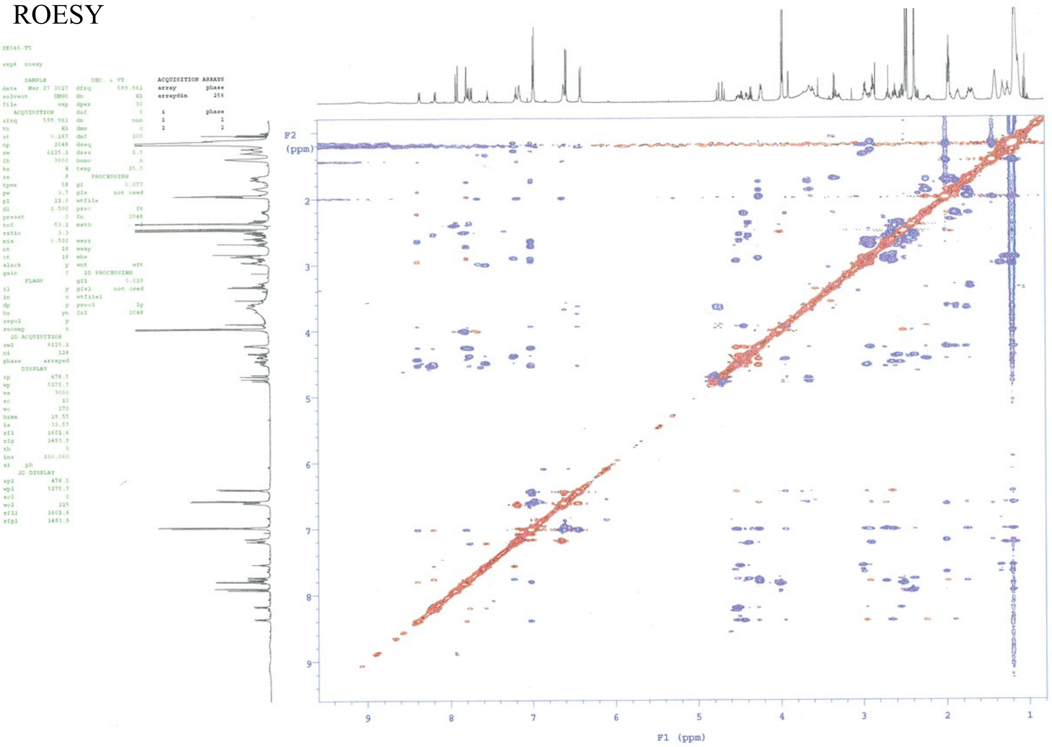

9. References

1) Imada, Y.; Iida, S.; Ono, S.; Masui, Y.; Murahashi, S. *Chem. Asian J.* **2006**, *1*, 136–147.

2) Ikeda, H.; Yoshida, K.; Ozeki, M.; Saito, I. *Tetrahedron Lett.* **2001** *42*, 252–2531.

3) Teunissen, J. P. A.; Paffen, F. E. T.; Ercolani, G.; de Greef, T. F. A.; Meijer, E. W. *J. Am. Chem Soc.* **2016**, *138*, 6852–6860.

4) (a) Hassner, A.; Dillon, J., Jr. *J. Org. Chem.* **1983**, *48*, 3382—3386; (b) Zhou, L.; Liu, X.; Ji, J.; Zhang, Y. Hu, X.; Lin, L.; Feng, X. *J. Am. Chem. Soc.* **2012**, *134*, 17023—17026.

5) Kaiser, E.; Colescott, R. L.; Bossinger, C. D.; Cook, P. I. *Anal. Biochem.* **1970**, *34*, 595.

6) Vojkovsky, T. *Pept. Res.* **1995**, *8*, 236.

7) Arakawa, Y.; Oonishi, T.; Kohda, T. Minagawa, K.; Imada, Y. *ChemSucChem* **2016**, *9*, 2769.

8) Imada, Y.; Iida, H.; Murahashi, S.-I.; Naota, T. *Angew. Chem. Int. Ed.* **2005**, *44*, 1704–1706.
